# Deep Learning-Assisted Organogel Pressure Sensor for Alphabet Recognition and Bio-Mechanical Motion Monitoring

**DOI:** 10.1007/s40820-025-01912-z

**Published:** 2025-09-08

**Authors:** Kusum Sharma, Kousik Bhunia, Subhajit Chatterjee, Muthukumar Perumalsamy, Anandhan Ayyappan Saj, Theophilus Bhatti, Yung-Cheol Byun, Sang-Jae Kim

**Affiliations:** 1 Nanomaterials & System Lab, Major of Mechatronics Engineering, Faculty of Applied Energy System, Jeju National University, Jeju, 63243 Republic of Korea; 2 Department of Computer Engineering, Jeju National University, Jeju-Si, 63243 Republic of Korea; 3 Department of Computer Engineering, Major of Electronic Engineering, Jeju National University, Institute of Information Science & Technology, Jeju, 63243 Republic of Korea; 4 Nanomaterials & System Lab, Major of Mechanical System Engineering, College of Engineering, Jeju National University, Jeju, 63243 Republic of Korea; 5 Research Institute of Energy New Industry (RINEI), Jeju National University, Jeju, 63243 Republic of Korea; 6 Interdisciplinary Graduate Program in Advanced Convergence Technology & Science, Jeju National University, Jeju, Republic of Korea; 7 Green Hydrogen Glocal Leading Research Center (gH2-RC), Jeju National University, Jeju, 63243 Republic of Korea

**Keywords:** Wearable, Organogel, Deep learning, Pressure sensor, Bio-mechanical motion

## Abstract

Wearable sensors integrated with deep learning techniques have the potential to revolutionize seamless human–machine interfaces for real-time health monitoring, clinical diagnosis, and robotic applications. Nevertheless, it remains a critical challenge to simultaneously achieve desirable mechanical and electrical performance along with biocompatibility, adhesion, self-healing, and environmental robustness with excellent sensing metrics. Herein, we report a multifunctional, anti–freezing, self-adhesive, and self-healable organogel pressure sensor composed of cobalt nanoparticle encapsulated nitrogen-doped carbon nanotubes (CoN CNT) embedded in a polyvinyl alcohol–gelatin (PVA/GLE) matrix. Fabricated using a binary solvent system of water and ethylene glycol (EG), the CoN CNT/PVA/GLE organogel exhibits excellent flexibility, biocompatibility, and temperature tolerance with remarkable environmental stability. Electrochemical impedance spectroscopy confirms near-stable performance across a broad humidity range (40%-95% RH). Freeze-tolerant conductivity under sub-zero conditions (−20 °C) is attributed to the synergistic role of CoN CNT and EG, preserving mobility and network integrity. The CoN CNT/PVA/GLE organogel sensor exhibits high sensitivity of 5.75 kPa^−1^ in the detection range from 0 to 20 kPa, ideal for subtle biomechanical motion detection. A smart human–machine interface for English letter recognition using deep learning achieved 98% accuracy. The organogel sensor utility was extended to detect human gestures like finger bending, wrist motion, and throat vibration during speech.

**Supplementary Information:**

The online version contains supplementary material available at 10.1007/s40820-025-01912-z.

## Highlights

- We rationally designed a robust, biocompatible CoN CNT/PVA/GLE organogel with self-healing, anti-freezing, and self-adhesive properties for wearable sensing applications.
- Incorporation of CoN CNT enables high-performance, stable pressure sensing for up to one month, with a sensitivity of S = 5.75 kPa^-1^, r^2^ = 0.978 in the detection range 0-20 kPa, with robust operation under high humidity and extreme temperatures (−20 to 45 °C).
- It accurately detects English alphabets, achieving 98% recognition accuracy using deep learning models.

**Supplementary Information:**

The online version contains supplementary material available at 10.1007/s40820-025-01912-z.

## Graphical abstract

**Figure:**
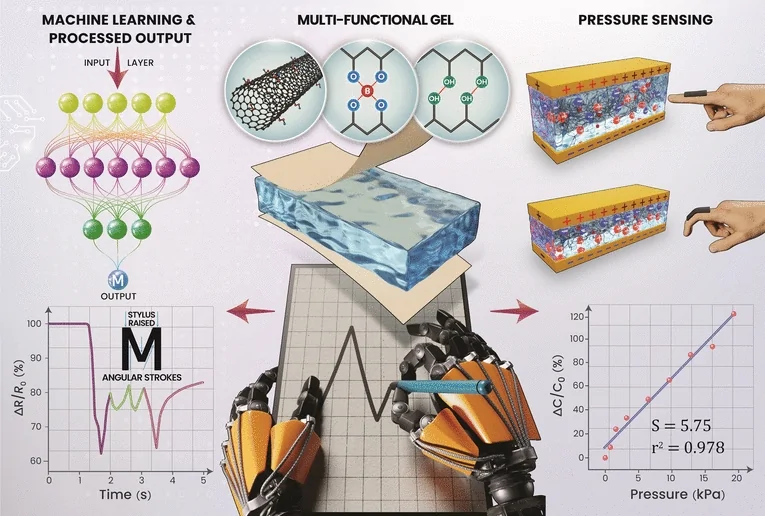

## Introduction

Driven by advances in skin-integrated electronics, wearable sensors have become essential in diverse applications, including real-time health monitoring, clinical diagnostics, human–machine interfaces and robotics [1–4]. Hydrogels, with their tissue-like softness, high water content, and biological affinity, have emerged as leading candidates. However, they suffer from key limitations such as dehydration, mechanical fragility, poor environmental stability, and limited conductivity, which hinders their long-term performance in real-world conditions [5]. To address these issues, researchers have introduced ionic salts, conductive polymers, carbon nanomaterials, and metal-based fillers into hydrogel matrices [6, 7]. For instance, Jiang’s team developed a conductive hydrogel by incorporating Mxene and explored the strategies to inhibit its oxidation, supported by density functional theory [8]. While these strategies enhance conductivity, they often compromise stability or biocompatibility and still fall short in achieving freeze resistance, self-healing, or sustained functionality under physiological conditions. Organogels have recently gained attention as promising alternatives, offering improved anti-freezing properties, dehydration resistance, and environmental durability while retaining softness and flexibility [9]. The use of binary solvents like ethylene glycol/water enhances ion mobility, polymer solubility, and crosslinking density, thereby improving mechanical integrity and electrical performance [10]. Notably, Tang et al. observed improved adhesion properties of the organogel using glycerin/water binary solvent compared to water as the only solvent for the same composition of the organogel [11]. Similarly, Su et al. explored sacrificial bonds between hydrogel matrix and glycerol via solvent displacement method to regulate the organohydrogel properties [12]. Consistent with recent efforts to integrate biodegradable and biocompatible materials into sensor platforms, the present organogel formulation embodies these principles via a PVA–gelatin and EG-based system. Although synthetic, PVA is water-soluble, non-toxic, and biodegradable under oxidative or enzymatic conditions, and has been FDA-recognized for usage in biomedical and dermal formulations [13]. Gelatin being biocompatible and biodegradable endows the gel matrix with abundant functional groups (-NH_2_, -COOH, -OH) and offers strong adhesion, biodegradability, and self-healing via reversible hydrogen bonding and triple-helix formation [14]. Together, these materials support the eco-friendly design and sustainable vision of the proposed wearable sensor platform.

Conventional gel-based pressure sensors are often hindered by low sensitivity, filler agglomeration, and poor environmental stability. To overcome these persistent limitations, we report for the first time, to the best of our knowledge —the incorporation of cobalt nanoparticle encapsulated nitrogen-doped carbon nanotubes (CoN CNT) into a PVA/gelatin/ethylene glycol (PVA/GLE/EG) organogel matrix for high-performance soft pressure sensing. This unique filler formulation represents a substantial advancement over conventional CNT doping strategies. Unlike pristine CNT or other carbon-based fillers, CoN CNT imparts multifunctional enhancements to the gel matrix. Specifically, the Co–Nₓ coordination sites induce strong interfacial polarization and facilitate efficient charge transport; nitrogen doping improves filler dispersion and enables hydrogen bonding with the polymer network, enhancing mechanical integration and stress dissipation; and the graphitic carbon shell provides a protective barrier against oxidation and hydrolysis of the Co core. Together, these synergistic features lead to enhanced dielectric modulation, electrical conductivity, and environmental robustness–culminating in exceptional pressure sensing performance not previously achieved with traditional fillers.

Organogel-based pressure sensors function via various transduction mechanisms, including piezoresistive, piezocapacitive, piezoelectric, and triboelectric effects [15–18]. Therefore, the selection of materials and structural design directly influences the sensing performance and device stability. For instance, Yao et al. designed a strain sensor using crack-engineered hydrogel fibers for gesture recognition, while Luo et al. developed a strain-insensitive e-skin with self-compensation via a chemically anchored island–bridge architecture [19, 20]. Further, a multi-modal e-skin based on hydrogel microstructure with self-calibration feature has been reported by Wang et al. [21]. To further enhance data processing and pattern recognition, deep learning techniques have been integrated with wearable/pressure sensors, to unlock the new possibilities for the development of intelligent human–machine interfaces [22–24]. The combination of machine learning and wireless systems has enabled real-time physiological monitoring and adaptive control [9, 25, 26]**.** By leveraging automatic feature extraction from resistive signals, deep learning algorithms enable high-precision classification of numerical and textual inputs, establishing a robust framework for smart sensing applications.

In this work, we present a deep learning-assisted CoN CNT/PVA/GLE organogel pressure sensor for intelligent human–machine interface applications. The developed organogel exclusively recognize English character with an accuracy of 98% validated by deep learning models. Time-series data obtained from the organogel sensor was classified by 1D (one-dimensional) convolutional neural networks (CNNs), long short-term memory (LSTM) networks, and XGBoost. A stacking classifier further validated the model’s accuracy, confirming the robustness of the approach. Additionally, the impact of writing speed and pressure variation on recognition accuracy was also investigated. The rationally designed CoN CNT/PVA/GLE organogel sensor meets the stringent requirements of next-generation wearable sensors by combining advanced material strategies with functional performance. The organogel exhibits self-healing behavior enabled by dynamic boronate ester bond formation, and strong surface adhesion through abundant hydrogen bonding interactions between the -OH groups of PVA and -NH_2_/-COOH groups of gelatin. The CoN CNT/PVA/GLE organogel demonstrates remarkable environmental stability, retaining its structural integrity and weight under ambient conditions for over 75 days. Its robust environmental adaptability was further evaluated through electrochemical impedance spectroscopy (EIS) across a broad temperature range (−20 to 60 °C) and relative humidity (RH) levels (40% to 95% RH). The organogel maintained a near-stable impedance response across varying humidity conditions, underscoring its resilience to moisture fluctuations. Importantly, under sub-zero conditions (−20 °C), the CoN CNT-doped organogel exhibited superior electrical conductivity compared to the pristine PVA/GLE counterpart, confirming its freeze-tolerant behavior. This enhanced performance is attributed to the synergistic effect of the CoN CNT conductive network and the anti freezing role of EG, which collectively preserves ion mobility and network integrity. Overall, this work introduces a multifunctional organogel platform that significantly advances the conventional design of hydrogel-based sensors. By combining robust material design with machine learning-driven intelligence, the system provides a comprehensive solution for wearable electronics, soft robotics, and personalized healthcare monitoring, offering reliable performance in dynamic, real-world environments.

## Experimental Section

### Materials

Polyvinyl alcohol (PVA) (high molecular weight, Sigma Aldrich), gelatin (GLE), sodium borate anhydrous (Na_2_B_4_O_7_), and ethylene glycol (EG), melamine, cobalt nitrate hexahydrate (Co(NO_3_)_3_.6H_2_O), ascorbic acid (C_6_H_8_O_6_), potassium hydroxide (KOH), and ethanol were purchased from Daejung Chemicals. All purchased chemicals were used as received without any further processing.

### Synthesis of CoN CNT, PVA/GLE and CoN CNT/PVA/GLE

#### Organogel Synthesis

A homogeneous mixture of PVA and GLE in a 1:1 weight ratio was prepared by dissolving the polymers in a binary solvent system comprising EG and deionized (DI) water. The mixture was continuously stirred and heated under controlled conditions until a translucent solution was obtained, indicating complete dissolution and uniform dispersion of the polymer chains. Separately, sodium borate anhydrous was dissolved in DI water and subjected to ultrasonic agitation for 1 h to ensure complete solubilization and uniformity. The sodium borate solution was then gradually added to the PVA/GLE solution, facilitating cross-linking through borate ester bond formation. The resulting solution was cast into molds and left to undergo physical gelation at room temperature, leading to the formation of a mechanically stable and robust organogel. For the synthesis of the CoN CNT/PVA/GLE organogel, CoN CNT were first dispersed in DI water using ultrasonication to achieve a uniform suspension. Subsequently, PVA and GLE in a 1:1 ratio was introduced into the CoN CNT dispersion and dissolved under the same controlled heating and stirring conditions. After complete dissolution of the polymer matrix, the pre-dissolved sodium borate solution was incorporated, and the final mixture was cast into molds. The system was then allowed to gel, forming a conductive, structurally reinforced organogel network embedded with CoN CNT. The synthesis procedure for CoN CNT and the characterization details have been provided in the Supplementary Information.

### Biocompatibility Test

#### Antibacterial Test

For the antibacterial assay, LB (Luria–Bertani) agar plates were inoculated with *Escherichia coli* BL21 (DE3) and incubated for 24 h. Organogel disks (8 mm diameter × 1 mm thickness) of pristine PVA/GLE and CoN CNT/PVA/GLE were prepared. Wells of the same dimensions were made in the agar, and the respective organogels were inserted. As experimental controls, ampicillin (10 µg mL^−1^) was used as a positive control and (phosphate-buffered saline) PBS as a negative control. To ensure reproducibility, the experiment was conducted on three independent plates, and the mean of their zones of inhibiting *E. coli* growth were recorded after Day1, 2, and 3. The plates were incubated at 37 °C to mimic physiological conditions.

#### Cytotoxicity Test

To specifically evaluate the cytotoxicity of CoN CNT, which are the primary component of concern, an MTT assay was conducted using RAW264.7 macrophage cells, following established protocols [27, 28]. Cells were seeded in 96-well plates (1.5 X 10^4^ cells/well) and exposed to various concentrations of CoN CNT (6.25, 12.5, 25, 50, and 100 µg mL^−1^) as shown in Fig. S35a-c. Lipopolysaccharide (LPS-EB; LPS from *E. coli* O111:B4) of concentration 1 µg mL^−1^ and untreated wells served as negative and positive controls, respectively. After 24 h of exposure, MTT reagent was added and incubated for 2 h. The resulting formazan crystals were then dissolved in DMSO, and the absorbance was measured at 540 nm using a Tecan SPARK plate reader.

### Statistical Analysis

For each measurement, three devices were prepared to ensure the reliability of the data. The error bar in various plots shows ± standard deviation for three sets of measurement taken on the prepared 3 devices.

### Deep Learning-Based Models and Validation

A detailed description of the deep learning models employed in this study–including 1D CNN, LSTM, XGBoost, and the final stacking classifier, is provided in Supporting Information (SI).

## Results and Discussion

### Organogel Design Strategy

**Fig. 1:**
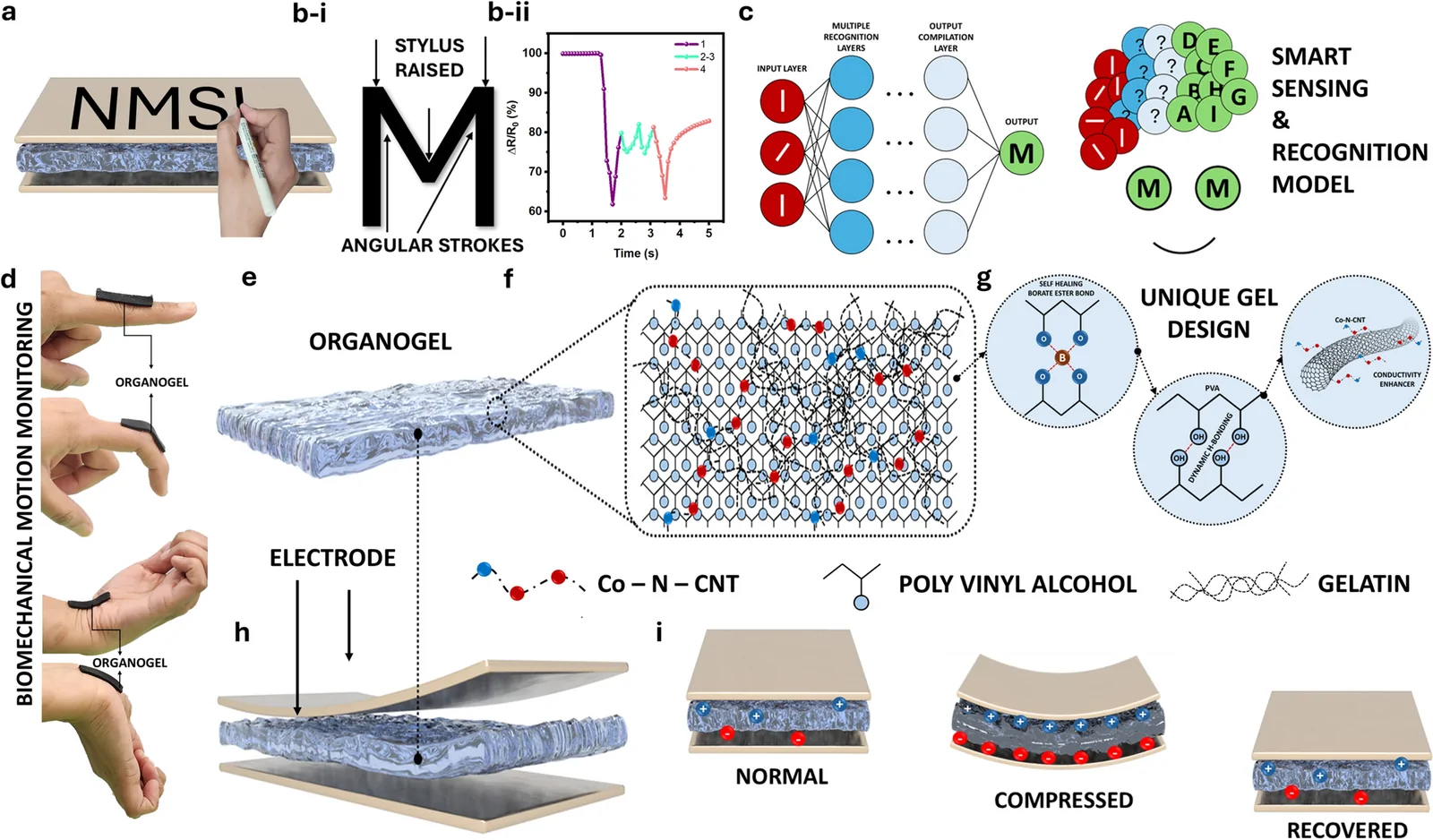
Overview and potential use cases of the CoN CNT/PVA/GLE organogel based pressure sensor. **a** Writing pattern on the organogel surface, showcasing its capability for precise spatial tracking. **b-i** Writing pattern of letter ‘M’ involving stylus movement, strokes etc. **b-ii** Electrical signal generation in response to the English letter 'M', demonstrating the sensor’s sensitivity to complex motion patterns. **c** Integration of deep learning models for capturing both spatial and temporal information, enhancing recognition accuracy. **d** Detection of subtle biomechanical movements, including finger and wrist bending, highlighting the organogel’s high flexibility and responsiveness. **e** Schematic representation of the organogel, with **f** an extended view: illustrating molecular interactions, **g** emphasizing the role of boronate ester bonds, dynamic hydroxyl bonding, and CoN CNT network in achieving optimal mechanical stability and dielectric properties. **h** Organogel-based pressure sensor device design. **i** Sensing mechanism based on piezocapacitive principles, detailing the dielectric modulation and capacitance variation under compressive forces

The design strategy and operational principle of CoN CNT/PVA/GLE organogel-based smart sensing and recognition system is illustrated in Fig. 1. The developed organogel demonstrates the capability to exclusively recognize handwritten English alphabets with an accuracy of 98%, as validated by deep learning models. Figure 1a–c provides a stepwise schematic representation of the entire process from writing on the organogel surface to signal generation and subsequent implementation of deep learning algorithms for alphabet recognition. Figure 1a demonstrates the initial step of writing on the organogel surface. Figure 1b-i presents the stylus-based writing process of the letter ‘M’, highlighting variations in angular and vertical strokes as well as stylus lift-offs, all of which contribute to a unique pressure signature for each letter.

Figure 1b-ii shows the corresponding resistive response generated during the writing of the letter ‘M’. Figure 1c depicts the deployment of multiple deep learning algorithms for the classification and recognition of handwritten English alphabets and alphanumeric characters based on time-series sensor data. In addition to alphabet recognition, the proposed system effectively detects subtle biomechanical signals, such as finger and wrist bending and throat vibrations, underscoring its potential in diverse human–machine interface applications (Fig. 1d). The schematic representation of the strategically designed CoN CNT/PVA/GLE organogel is presented in Fig. 1e, highlighting its structural layout and functional architecture. A magnified view of a selected region of the organogel is shown in Fig. 1f, illustrating the molecular-level arrangement within the gel matrix. Furthermore, Fig. 1g delineates the key molecular interactions responsible for the unique physicochemical and functional characteristics of the organogel. These include hydrogen bonding, borate ester linkages, and conductive network formation facilitated by CoN CNT, all of which synergistically contribute to the gel’s mechanical robustness, self-adhesive behavior, and superior sensing performance. Together, PVA and gelatin provide a biocompatible, flexible, and adhesive matrix with tunable mechanical properties. To further optimize its functionality, we employed an EG/water binary solvent system, which imparts multidimensional enhancements to the organogel such as anti-freezing properties, improved ionic conductivity, thermoplasticity, and mechanical durability by modulating hydrogen bonding and facilitating better polymer–solvent interactions. To further elevate electrical conductivity and mechanical robustness, we introduce CoN CNT into the PVA-gelatin organogel matrix. Unlike conventional CNT, CoN CNT provide enhanced electron transport due to the metallic Co core and defect-rich nitrogen doping. These features also improve mechanical interlocking within the gel matrix and facilitate both ionic and electronic conduction. This hybrid conductive network ensures reliable performance even under extreme temperatures, addressing a key limitation of existing hydrogel-based systems. The sensor works on piezocapacitive transduction mechanism, where the mechanical deformation has been converted into electrical signals (Fig. 1h-i). The developed organogel-based sensor demonstrates significant potential in soft robotics, personalized healthcare monitoring, medical diagnostics, and human–machine interaction application.

### Physicochemical Analysis

**Fig. 2:**
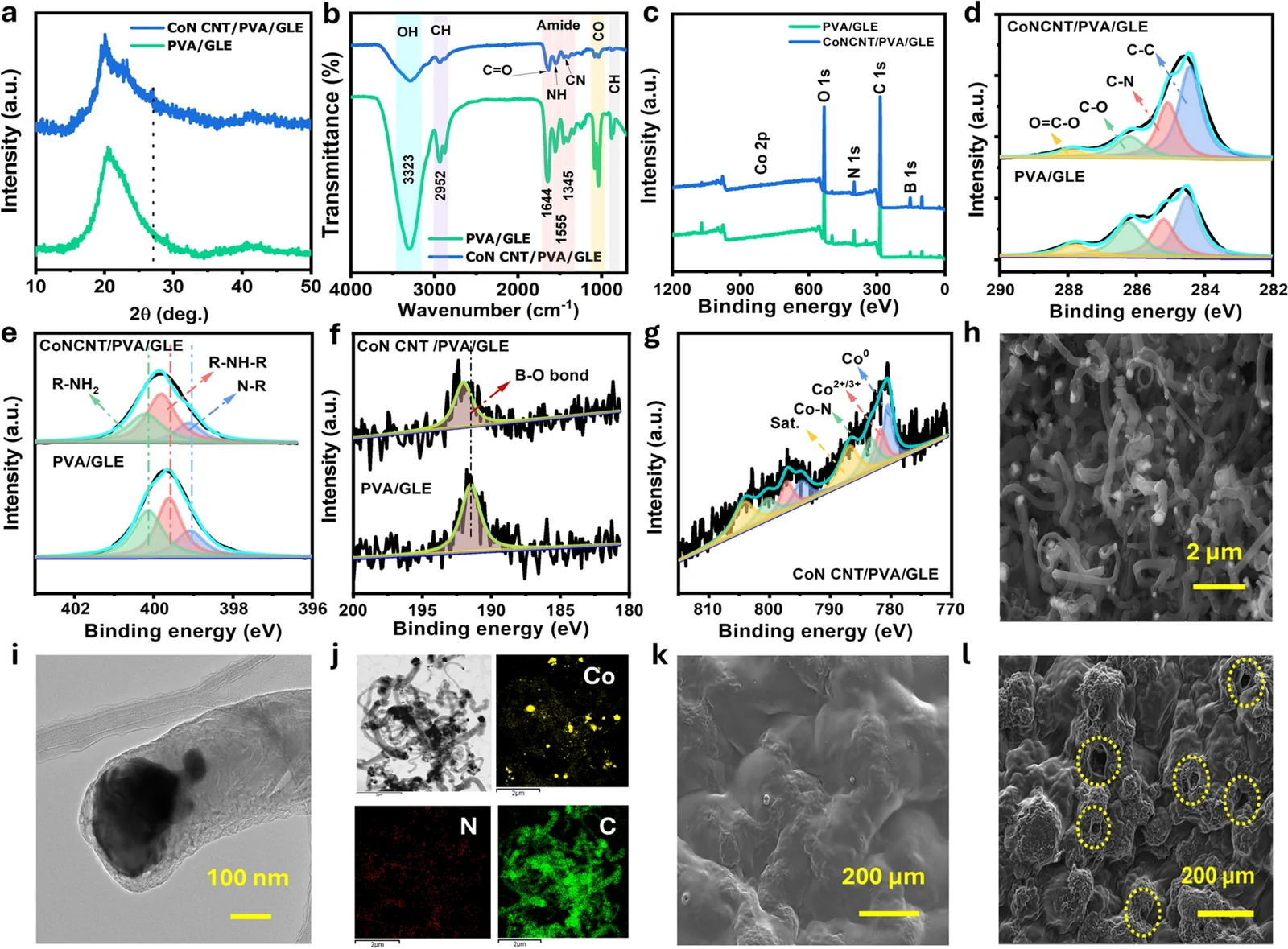
Physiochemical characterization of the organogel. **a** XRD pattern, **b** FTIR, and **c** XPS survey spectra of PVA/GLE and CoN CNT PVA/GLE organogel respectively. High resolution spectra of **d** C 1*s*, **e** N 1*s*, **f** B 1*s* for PVA/GLE and CoN CNT PVA/GLE organogel. **g** High resolution XPS spectra of Co 2*p* in CoN CNT PVA/GLE organogel. **h** FESEM image of CoN CNT. **i** TEM image of CoN CNT, and **j** respective elemental mapping. FESEM images of **k** PVA/GLE and **l** CoN CNT/PVA/GLE organogel

The XRD of PVA/GLE and CoN CNT/PVA/GLE organogels are shown in Fig. 2a. The PVA/GLE organogel exhibited broad peaks around 20° and 40°, characteristic of PVA, indicating the amorphous phases of both PVA and GLE. The incorporation of CoN CNT into the PVA/GLE matrix was confirmed by a distinct diffraction peak at 26.8°. The powder XRD pattern of pure CoN CNT is shown in Fig. S1a. The diffraction peak at 26.5° corresponds to the (002) plane of graphitic carbon. Additional peaks at 44.2°, 51.5°, and 75.6° correspond to the (111), (200), and (220) planes of the face centered cubic phase of metallic Co nanoparticles (JCPDS 89–4307) [29]. Furthermore, the XRD patterns of pure PVA and GLE powders are presented in Fig. S1b. The FTIR spectroscopy was used to investigate intermolecular interactions in PVA/GLE organogels with and without CoN CNT, as displayed in Fig. 2b. The vibration band observed at 3323 cm^−1^ was ascribed to the combined O–H/N–H (amide-I) bond and intramolecular hydrogen bond vibrations; whereas the vibration bands positioned at around 2952, 2884, 1644, and 1555 cm^−1^_,_ correspond to C-H stretching, acetyl C = O groups, and N–H (amide II) bond vibration [30]. In addition, the vibration bands shifted to lower wavenumbers (1644 to 1633 cm^−1^ and 1555 to 1549 cm^−1^) for the CoN CNT/PVA/GLE indicating interactions between CoN CNT and the PVA/GLE matrix, primarily through weak van der Waals forces. This shift reflects a modification in the local environment around the carbonyl groups, suggesting partial charge delocalization or altered electronic density due to the incorporation of CoN CNT [31]. Additionally, the vibrational band at 1345 cm^−1^ confirms the formation of boronic ester bonds within the organogel structure, further reinforcing crosslinking and network stability. Boronic ester bonds likely enhance mechanical robustness by reducing segmental mobility, contributing to increased strength and elasticity. Further, the FTIR spectra of pure PVA, GLE powder, and EG have been provided in Fig. S2.

XPS analysis was carried out to further elucidate the chemical environment and surface interactions within the organogel matrix. The survey spectra of PVA/GLE organogel in the presence and absence of the CoN CNT has been displayed in Fig. 2c, confirming the presence of boron (B) along with C, N, O, and Co in the CoN CNT/PVA/GLE organogel. The survey spectra, as well as high-resolution core-level spectra C 1*s*, N 1*s*, and Co 2*p* of the as-synthesized CoN CNT, is shown in Fig. S3. The deconvoluted C 1*s* spectra indicates the presence of the C = C bond (284.5 eV), C-N bond (284.9 eV), and C-O bond (285.83 eV) in CoN CNT (Fig. S3b). Similarly, the deconvoluted N 1*s* spectra indicate the presence of pyridinic-N (398.67 eV), pyrrolic-N (400.17 eV), graphitic-N (401.22 eV), and oxidized nitrogen (403.59 eV) moieties in the CoN CNT (Fig. S3c) indicative of successful nitrogen doping in CNT.

The deconvoluted Co 2*p* spectra described the coexistence of the metallic Co^0^ state (778.67 eV) along with Co^2+/3+^ (779.88 eV) state and Co–N bond (781.39 eV) formation (Fig. S3d). The presence of metallic cobalt and Co–N interactions, confirming successful encapsulation Co nanoparticles in the nitrogen doped CNTs. The formation of Co-N_x_ coordination within the conductive carbon matrix is particularly significant for pressure sensing, as it facilitates interfacial polarization and charge accumulation at the filler–polymer interface. These effects enhance the dielectric responsiveness of the system under applied pressure. Moreover, the graphitic carbon framework ensures high electron mobility and mechanical robustness, essential for stable sensor operation. The high-resolution spectra of C 1*s* for both the organogels (PVA/GLE and CoN CNT/PVA/GLE) is shown in Fig. 2d. The deconvoluted C 1*s* spectra of the organogel indicates the presence of the C = C bond (284.5 eV), C-N bond (285.19 eV), C–O bond (286.19 eV), and O = C–O type bond (287.88 eV) arises from the functional groups present in the polymer chain of PVA and GLE [32]. The high-resolution spectra of N 1*s* of the PVA/GLE has been deconvoluted into three binding energy regions, indicating the coexistence of the N-R (399.04 eV), secondary amine (399.59 eV), and primary amine group (400.13 eV) of the GLE (Fig. 2e). The binding energy of N 1*s* shifted to a higher value in the CoN CNT/PVA/GLE organogel. This shift indicates electron density delocalization within the organogel due to the incorporation of CoN CNT [33]. Figure 2f shows the XPS spectrum of B 1*s*. The binding energy peak at 191.47 eV for the PVA/GLE organogel confirms the formation of boronate ester crosslinked PVA. Upon incorporation of CoN CNT, the B 1*s* binding energy shifts to a higher value, suggesting modification in the local environment of the boronate ester bond [34]. The deconvoluted spectra of O 1*s*, indicating the presence of C-O, C = O, and C–O–H/B bonding has been displayed in Fig. S4. Similar to N 1*s*, the binding energy region is shifted to higher energy upon incorporation of CoN CNT in the PVA/GLE matrix. The XPS Co 2*p* of the CoN CNT/PVA/GLE is presented in Fig. 2g. The Co 2*p* XPS revealed the coexistence of the metallic Co^0^ state (780.31 eV), surface oxidized Co^2+^/Co^3+^ (781.58 eV), and Co–N bond formation (783.16 eV). However, the binding energy of the different oxidation state species of the Co 2*p* of CoN CNT present in the PVA/GLE is shifted to higher energy compared to bare CoN CNT. The morphology of the synthesized CoN CNT was examined using FESEM images as shown in Figs. 2h and S5. A tubular structure was clearly observed, with bright spots at the center corresponding to Co nanoparticles. HRTEM images (Fig. 2i) further confirm the successful encapsulation of Co nanoparticles within the graphitic CNT structure. Moreover, the corresponding elemental mapping (Fig. 2j) reveals a uniform distribution of Co, N, and C elements throughout the synthesized composite. The surface morphology of the pristine PVA/GLE is shown in Figs. 2k and S6, revealing a coarse and uneven surface. Notably, as shown in Fig. 2l the CoN CNT/PVA/GLE sample exhibits significantly increased surface roughness with macropores (encircled region). Cross-sectional views of both hydrogels are displayed in Fig. S7 and S8, respectively.

### Mechanical Performance of the Organogel

**Fig. 3:**
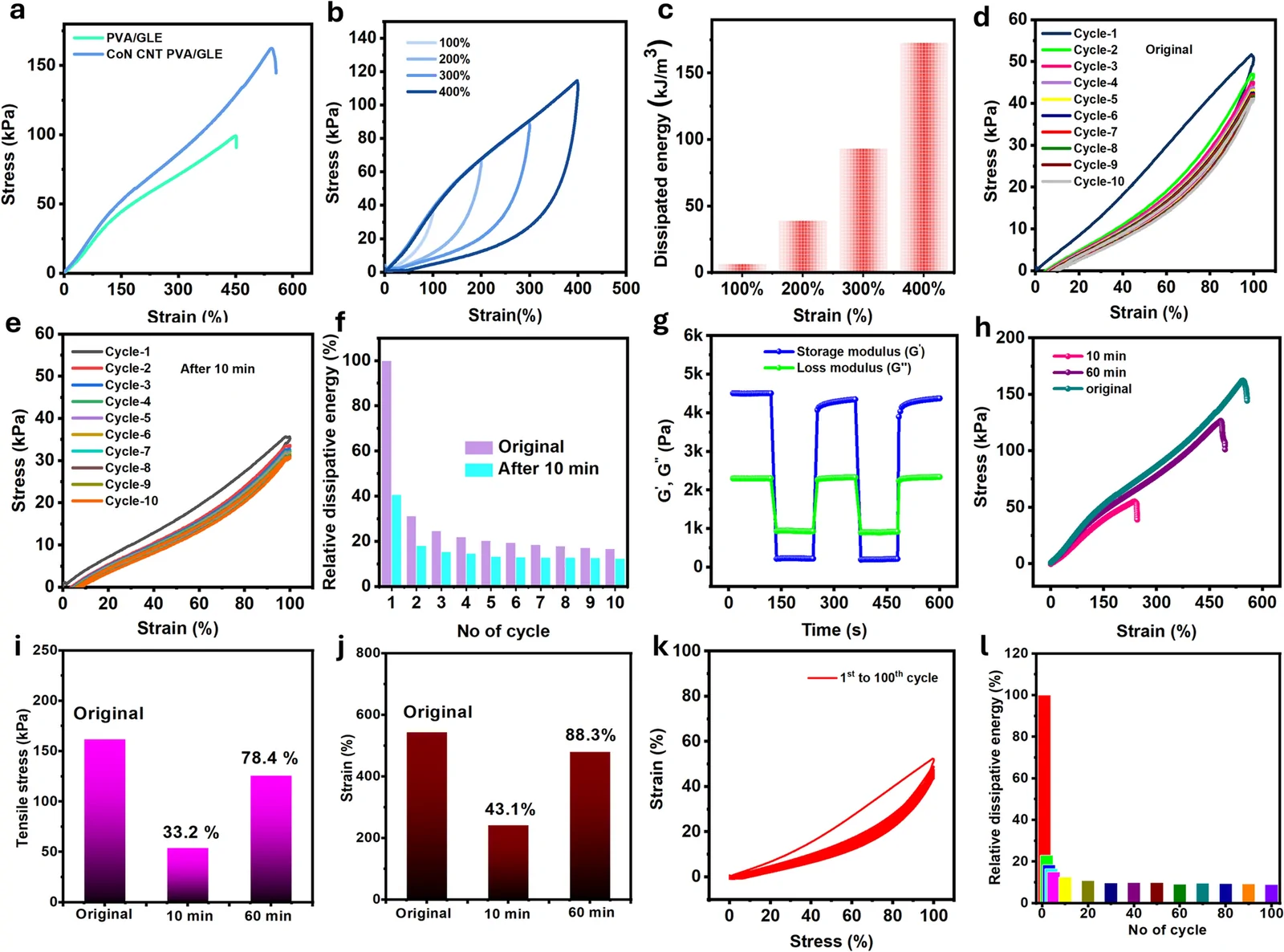
Mechanical properties of organogel.** a** Stress–strain profile of the organogel, **b** cyclic loading–unloading test at different strain level for CoN CNT/PVA/GLE organogel, **c** dissipated energy at different strain level, **d** loading–unloading test for ten consecutive cycles without rest, **e** consecutive stress–strain cyclic response after 10 min of rest, **f** calculated dissipated energy profile for each cycle: before and after 10 min of rest for the CoN CNT/PVA/GLE organogel. **g** G′ and G″ of the cyclic steps with shear strain shifting between 1 and 200%, **h** tensile stress–strain profile after different healing time, **i-j** stress and strain recovery percentage with time, **k** 100 cycles loading–unloading test without rest, and **l** corresponding dissipative energy retention plots

The mechanical properties of the synthesized organogel are critical for its application in wearable devices. To evaluate its tensile behavior, a comprehensive set of mechanical tests was performed. The tensile stress–strain curve, displayed in Fig. 3a, reveals a marked improvement in toughness and strain capacity with the incorporation of CoN CNT. The tensile strength increased from 107.58 ± 6.62 to 144.51 ± 14.55 kPa, alongside a slight rise in elastic modulus from 28.46 ± 1.26 to 29.13 ± 3.85 kPa. The high stretchability and toughness of the CoN CNT/PVA/GLE organogel can be summarized by the combined effect of CoN CNT nano-reinforcement and non-covalent interactions, which include suitable hydrogen bonds between GLE and PVA, borate esterification of PVA chain. Cyclic loading/unloading tests at various strain levels is shown in Fig. 3b, indicating a progressive increase in hysteresis with higher strain percentages. The dissipated energy calculated from these cyclic plots are displayed in Fig. 3c, being 20 kJ m^−3^ at 100% strain to become 175 kJ m^−3^ at 400% strain. To probe the mechanical durability of the CoN CNT/PVA/GLE organogel, anti-fatigue experiments were performed at 100% strain over 10 repetitive cycles without rest time (Fig. 3d). After the first cycle, the organogel is unable to regain its original dissipated energy due to the lack of resting during the successive loading–unloading test. Thereafter, the organogel is rested for 10 min, and successive ten loading–unloading cycles were performed (Fig. 3e). The organogel partially regained its mechanical properties, with the relative energy dissipation (RED) 12%±0.56% check at 12 ± 0.56% across subsequent cycles (Fig. 3e). The anti-fatigue test results are summarized in Table TS1 (SI), while Fig. 3f quantitatively illustrates the recovery and energy dissipation for each cycle, showcasing the hydrogel's resilience and capacity for mechanical recovery.

The self-healing behavior of the synthesized CoN CNT/PVA/GLE organogel was evaluated using rheological measurements. As shown in Fig. 3g, the storage modulus (G') was significantly higher than the loss modulus (G") at low strain (γ = 1%), indicating the formation of a self-standing organogel. However, when subjected to high strain amplitude (γ = 200%, frequency = 1.0 Hz), both G' and G" values dropped drastically, suggesting a breakdown of the internal network structure. The substantial reduction in G' relative to G" reflects a transition from a quasi-solid to a quasi-liquid state, indicating integrity loss due to internal fracture. Upon returning the strain to 1%, the G' and G" values rapidly recovered to their original levels, demonstrating the organogel’s excellent self-healing capability. This recovery was reproducible over three consecutive strain cycles, confirming material’s robust self-recovery behavior. The tensile stress–strain behavior of the organogel after different healing durations is presented in Fig. 3h. Healing times of 10 min, and 60 min were selected for evaluation. After 10 min of healing, the tensile strength and maximum strain recovered to 33% and 43% of their original values, respectively.

After 60 min, these values increased to 78% and 88.2%, as shown in Fig. 3i. The corresponding tensile strain recovery profiles are displayed in Fig. 3j. Furthermore, successive loading–unloading tests were performed to assess the long-term durability of the organogel. A total of 100 continuous loading–unloading cycles were conducted. After 100 cycles, the organogel retained approximately 9% of its initial mechanical performance, as illustrated in Fig. 3k, l.

**Fig. 4:**
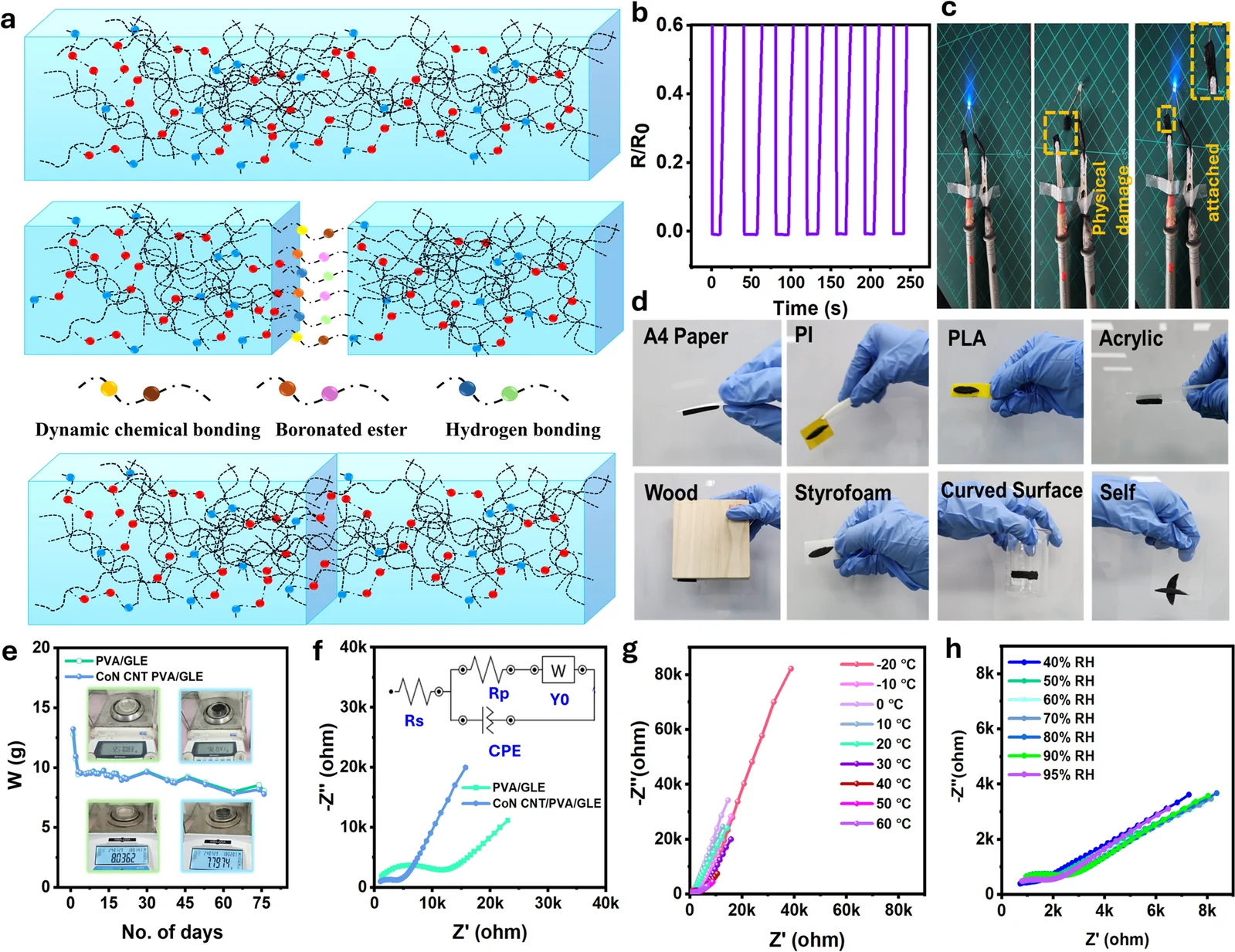
**a** Schematic representation of the self-healing mechanism. Electrical self-healing test of the organogel,** b** resistance variation upon cutting and attaching the organogel, exhibiting its self-healing nature, **c** respective digital images upon cut and attach. **d** Adhesive behaviour of the organogel on various substrates. **e** Weight loss measurement of the organogel for 75 Days. **f** Nyquist plot for PVA/GLE and CoN CNT/PVA/GLE organogel at 30 ºC and 40% RH. Nyquist plot for CoN CNT/PVA/GLE organogel under varying **g** temperature from -20 ºC to 60 ºC,** h** relative humidity (40% RH-95% RH)

A schematic representation of the self-healing mechanism has been included Fig. 4a, highlighting the dynamic reformation of boronic ester bonds and restoration of percolated conductive paths through CoN CNT, supported by secondary hydrogen bonding and van der Waals interactions. This dynamic bonding hierarchy enables both mechanical and electronic healing. The electrical self-healing behavior of the CoN CNT/PVA/GLE organogel was systematically investigated by examining its resistance recovery after physical damage. The intrinsic self-healing capability stems from the presence of dynamic and reversible bonding interactions within the gel matrix. A rectangular strip of the organogel was cut completely and then manually rejoined without applying any external stimuli such as heat, humidity, or light. The experiment was conducted under ambient laboratory conditions (~ 25 °C and 45%-50% RH). Upon cutting, the electrical resistance rose from its base value (~ 95 kΩ) to the maximum measurable resistance (~ 22 GΩ), indicating an open circuit condition (Figs. 4b and S9). Once reattached, the resistance instantly dropped back to its original value within 0.24 s, confirming autonomous and instantaneous healing (Fig. S10). This rapid recovery indicates effective reformation of the boronic ester crosslinks and hydrogen bonds, which restore both structural cohesion and conductive pathways via the embedded CoN CNT network [27, 28]. To validate the durability and reproducibility of self-healing behavior, the cut-and-heal process was repeated multiple times. In each cycle, the resistance returned to its original value, confirming consistent electrical recovery and repeatable network reconstruction (Movie S1). To demonstrate the functional implications of this self-healing behavior, a closed-loop circuit was formed with the organogel connected to a light emitting diode (LED) (Fig. 4c). When the gel was cut, the LED turned off. Upon rejoining, the LED immediately glowed again, verifying complete restoration of electrical conductivity and confirming practical self-healing capability (Movie S2). Moreover, the gel was tested under extreme conditions by storing it at −20 °C for 48 h and repeating the cut-and-heal experiment. Remarkably, even under these sub-zero conditions, the gel maintained its healing performance. The LED circuit test and mechanical reconnection (Fig. S11, Movies S3 and S4) confirmed full recovery, attributed to the stability of boronic ester and hydrogen bonds at low temperatures. Furthermore, the adhesive behavior of the synthesized organogel was tested on various substrates, which include human skin, a ceramic substrate, paper, wooden pieces, and so on, as digital images presented in Fig. 4d. The robust adhesion nature of the CoN CNT/PVA/GLE organogel is primarily correlated to the combined effect of hydrogen bonding, covalent crosslinking, *π-π* interactions, and metallic coordination involving hydroxyl, amine, carboxyl, and oxidized groups within the matrix and on the CNT surface. Further, the adhesion towards other surfaces and at various angles has been provided in Figs. S12-S14.

As organogel physiochemical properties are greatly influenced by environmental factors such as temperature and humidity, further its application in wearable pressure sensing necessitates studying the effect of environmental factors on its stability. The weight of the organogel, kept in open condition, has been meticulously monitored for more than two months, along with ambient temperature and humidity. Figure 4e shows the plot for variation of weight of organogel over 75 Days. Initially, a significant weight loss of 2% was observed for the 2nd and 3rd Day, respectively. Thereafter, no significant weight loss was observed for more than 75 Days. This stability is attributed to the use of a binary solvent system, carefully optimized in composition and ratio, which effectively prevents dehydration and contributes to the organogel's durable, application-ready performance. The digital images showing the variation of PVA/GLE and CoN CNT/PVA/GLE organogel, along with humidity & temperature, have been displayed in Figs. S15-S22.

### Electrical Performance Analysis

The electro-responsive behavior/nature of the organogel has been analyzed using EIS. Figure 4f shows the Nyquist plot of the PVA/GLE organogel (green color) and CoN CNT/PVA/GLE organogel (blue color) at 30 °C and 40% RH. The large semicircle for PVA/GLE organogel reflects its higher overall impedance (series resistance, R_s_ = 850 Ω and polarization resistance, R_p_ = 10.1 kΩ). On the other hand, CoN CNT incorporated organogel shows a dramatically smaller semicircle and lower overall impedance (R_s_ = 858 Ω and, R_p_ = 2.63 kΩ), signaling enhanced charge transfer. The incorporation of CoN CNT introduces highly conductive channels that lower the charge transfer resistance. These nanotubes act as bridges for electron movement, facilitating more efficient electron flow across the organogel matrix. Additionally, the improved linear region at lower frequencies indicates better ion diffusion and reduced polarization effects, suggesting that the material handles both electron and ion transport with greater efficiency. To further analyze the electrical behavior of CoN CNT/PVA/GLE organogel, under varying temperature and humidity conditions, the EIS measurements were carried out from −20 to 60 °C as shown in Fig. 4g. The temperature-dependent EIS analysis reveals the superior conductive performance of the CoN CNT/PVA/GLE organogel (1.10 mS cm^−1^ at -20 °C) compared to pristine PVA/GLE (75.29 μS/cm), particularly under sub-zero conditions (Fig. S23). At −20 °C, CoN CNT/PVA/GLE exhibits markedly lower R_s_ = 56.1 Ω and R_p_ = 544 Ω, while pristine PVA/GLE shows significantly higher R_s_ (850 Ω) and R_p_ (118 kΩ). This pronounced contrast highlights the role of CoN CNT in enhancing charge transport and maintaining conductivity in frozen environments. Additionally, the relatively low constant phase element (CPE) values and a high CPE exponent (N ≈ 1.1) for CoN CNT/PVA/GLE indicate minimal deviation from ideal capacitive behavior and stable dielectric properties. Conversely, PVA/GLE displays a gradual decrease in R_p_ at elevated temperatures, reflecting poor conductivity in cold conditions. The EIS measurements under varying relative humidity (RH) conditions for the CoN CNT/PVA/GLE organogel are presented in Fig. 4h, with comparative data for the PVA/GLE organogel shown in Fig. S24. For the CoN CNT/PVA/GLE system, R_s_ and R_p_ exhibit only minor changes across the RH range of 40%–95%, with R_s_ increasing modestly from 642 Ω at 40% RH to 857 Ω at 70% RH, a variation of approximately 200 Ω. The CPE also remains relatively stable at around 0.3 nMho·s^N^, indicating minimal impact of humidity on charge transport and dielectric properties. This suggests that the CoN CNT/PVA/GLE organogel possesses excellent environmental stability and robustness under humid conditions. In contrast, the PVA/GLE organogel shows a pronounced sensitivity to humidity, with R_s_ dropping sharply from 835 Ω at 40% RH to 80 Ω at 95% RH, and R_p_ decreasing from 12 to 0.82 kΩ over the same range. Additionally, there is a substantial increase in both CPE and Warburg elements, indicating enhanced ionic conductivity and diffusion at higher humidity levels. The CPE exponent (N) also declines from 0.788 at 40% RH to 0.479 at 90% RH, reflecting a significant deviation from ideal capacitive behavior (Table TS2, and TS3 in SI).

### Pressure Sensing Performance

**Fig. 5:**
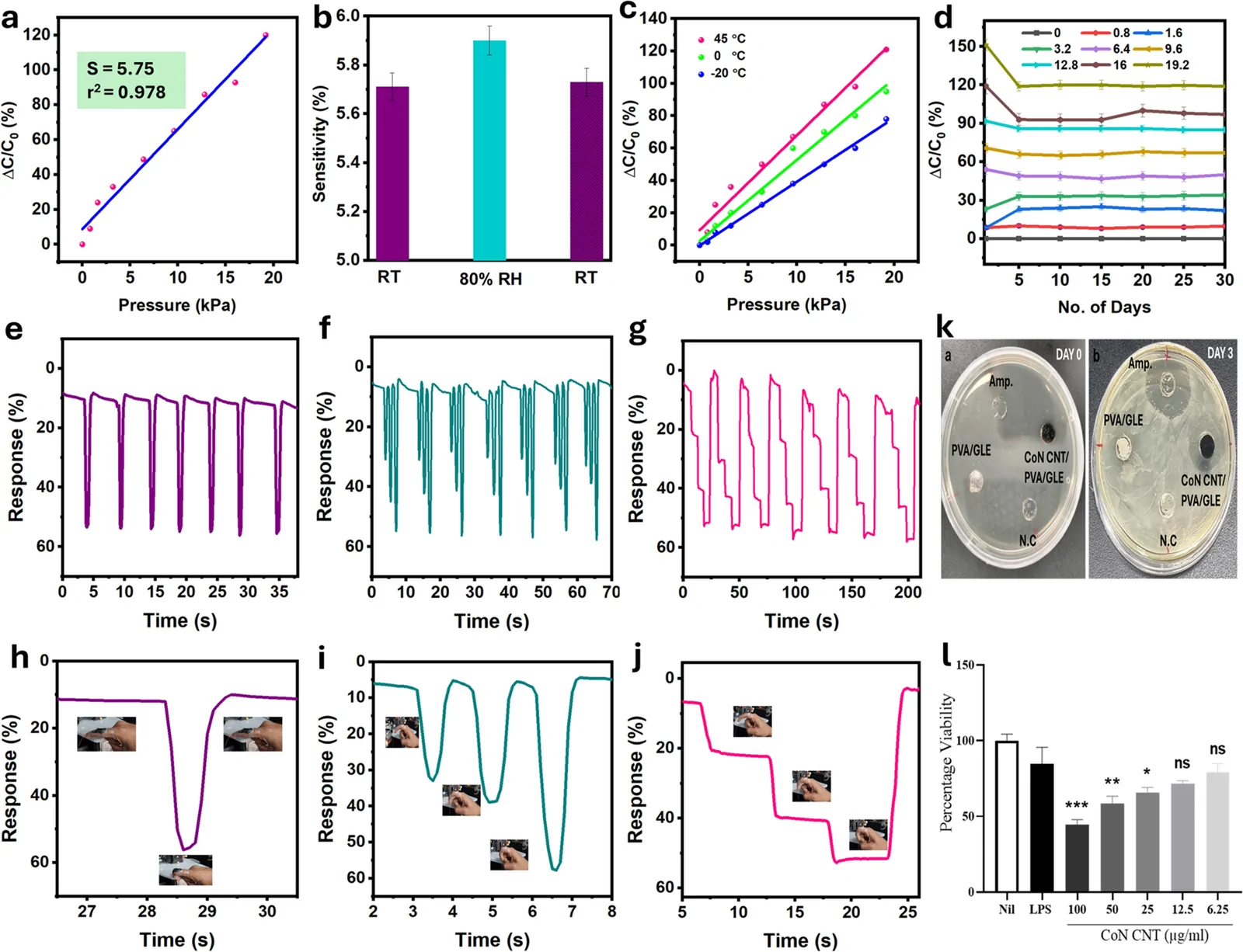
**a** Capacitive pressure response characteristic in the detection range 0—20 kPa,** b** drift in the sensitivity after storing the gel at 80% RH level for 48 h,** c** pressure sensing plot at 45 °C, 0 °C, and -20 °C, **d** long term stability plot. Real time bio-mechanical motion monitoring of** e** finger bending,** f** bending at different angles,**g** bending at different angles with holding for: 5 s at each bending position, **h-j** respective magnified view: exhibiting single cyclic response (Fig. e–g). **k** Anti-microbial testing of CoN CNT/PVA/GLE up to 72 h against *E. coli bacteria*. For negative control (N.C) PBS, and for positive control ampicillin (Amp.) (10 µg/ml) was used for Day 0 and Day 3 of application, respectively. **l** Cell viability of different concentrations of CoN CNT on RAW264.7 cells. Cell viability was assessed using MTT assay at 24 h of cell treatment with CoN-CNT. CoN-CNT viability values are compared with that of LPS. All values are means ± SD, n = 3. *p < 0.01, **p < 0.001, ***p < 0.0001

The sensor comprises an organogel layer sandwiched between two conductive electrodes, forming a parallel-plate capacitor (Fig. 1h). The organogel sensing layer was rationally engineered to integrate mechanical adaptability with enhanced dielectric and conductive functionalities. The dual-polymer matrix, comprising PVA and GLE, forms an interconnected, physically crosslinked network through dynamic hydrogen bonding and reversible borate ester linkages. This architecture imparts the organogel with elasticity, resilience, and rapid mechanical recovery, ensuring reliable response under cyclic loading. Plasticization with a binary solvent system of EG and DI water enhances polymer chain mobility and ionic conductivity, facilitating fast dielectric relaxation and dynamic dipole reorientation during deformation. To augment electronic functionality, CoN CNT was incorporated to form percolated conductive networks (Fig. S25). The synergistic effects of metallic cobalt core and nitrogen doping significantly enhance charge transport and interfacial polarization, contributing to signal stability and sensitivity [35]. The resulting pressure sensor operates via a piezocapacitive mechanism, wherein applied pressure induces a marked increase in capacitance through three concurrent pathways: (i) reduction in inter-electrode distance due to compression of the soft organogel matrix, (ii) reconfiguration and densification of the CoN CNT conductive network, and (iii) modulation of the effective dielectric constant via polymer chain rearrangement, solvent redistribution, and dipolar alignment [36]. The capacitance, as governed by the equation:

1 $$C= \frac{\in .A}{d}$$

where, ∈ is the relative permittivity, A is the area of the electrodes and d is the thickness of the organogel. As pressure increases, the organogel densifies, leading to a measurable capacitance change, which is then correlated to the applied force. The pressure sensing plot has been provided in Fig. 5a in the range 0 to 20 kPa, showing a sensitivity of 5.75 kPa^−1^ with a linearity coefficient of r^2^ 0.978. The sensitivity has been calculated using the formula:

2 $$S= \frac{\Delta C/{C}_{o}}{\Delta P}$$

where ΔC is the change in capacitance, ${C}_{o}$ is the base capacitance, i.e. the capacitor without any stimulus/pressure; ΔP is the respective change in pressure. For subtle pressure measurement caused mainly by body movements, possessing good sensitivity in the low-pressure range is very crucial.

The influence of humidity on pressure sensing performance was evaluated by recording the sensor response, at RH levels of 60% and 80% (Fig. S26a-c). The sensor exhibited negligible variation in sensitivity, with values of 5.78 ± 0.34 and 5.85 ± 0.30 at 60% and 80% RH, respectively. To assess long term stability under humid conditions, the sensor was exposed to 80% RH for 48 h, during which it maintained a consistent sensitivity of 5.90 ± 0.30 slightly higher than the value under ambient conditions (5.75). However, upon returning to ambient conditions, the sensitivity fully recovered (5.75), confirming excellent environmental resilience (Fig. 5b). This robustness is attributed to the binary solvent system (EG/DI water) and borate-mediated crosslinking, which enhance hydrogen bonding stability and suppress water uptake [9, 37]. To investigate the temperature tolerance, the sensor performance was measured at 45, 0, and − 20 °C, as shown in Figs. 5c and S27a-f. Sensor performance remained stable at 45 and 0 °C with sensitivities of 5.85 ± 0.31 and 5.73 ± 0.30, respectively (< 0.2% variation). At − 20 °C, a reduced sensitivity (3.94 ± 0.08) was observed due to matrix stiffening. However, the sensor still responded reliably under low pressure. Upon returning to room temperature, both mechanical flexibility and sensing performance were fully restored, confirming excellent reversibility (Figs. S28 and S29).

The dynamic borate crosslinks and anti-freezing binary solvent system collectively ensure stable operation across 40–80% RH and −20 to 45 °C, outperforming typical hydrogels that often fail under such conditions [38–40]. Compared to conventional hydrogels, which typically suffer from freezing-induced brittleness and evaporation-driven instability, the designed organogel offers superior durability and long-term stability. To further assess the durability and reliability of the sensor, its performance was monitored over a period of 30 days. Capacitive responses were recorded at 5-day intervals and are presented in Fig. S30a-g. On Day 1, the sensitivity was measured at 7.62 ± 0.20, which stabilized to 5.75 ± 0.29 from Day 5 through Day 30, showing a minimum variation of less than 1%. All measurements exhibited excellent linearity, with correlation coefficient (r^2^) consistently exceeding 0.95. A detailed summary of the sensitivity values is provided in Table TS4. Figure S30h presents a comparative capacitive response across the 30-day period, while Fig. 5d shows the corresponding stability plot. The sensor demonstrated consistent performance throughout the monitoring period, highlighting the inherent durability of the organogel matrix-a property often lacking in conventional gel-based sensing platforms. This long-term stability is primarily attributed to the chemically robust design of the organogel network and the encapsulation of Co nanoparticles within a graphitic carbon shell, which preserves structural integrity and supports sustained electrical performance [35]. Additionally, the presence of mobile ionic species within the gel matrix contributes to stable ionic conductivity over time. These findings strongly support the long-term operational reliability and robustness of the proposed sensor architecture.

While many flexible pressure sensors demonstrate high sensitivity, their real-world application is often hindered by limited environmental resilience, poor long-term stability, or lack of self-repairing capabilities. In contrast, the CoN CNT/PVA/GLE-based sensor developed in this study successfully integrates high sensitivity (5.75 kPa^−1^), operational range (0–20 kPa), and mechanical compliance, with rare features such as temperature/humidity tolerance and autonomous healing. Notably, stable performance was maintained over cyclic loading and 30-days ambient exposure, underscoring its durability. Moreover, the sensor’s self-healing capability was demonstrated both electrically (instant recovery) and mechanically (88% stress–strain recovery), reflecting a synergistic material design. These attributes, combined with biocompatibility and ambient robustness, distinguish the present platform from existing gel-based sensors and support its candidacy for next-generation wearable electronics. A comparative overview of key parameters is summarized in Table 1.

**Table 1:** Performance comparison of the CoN CNT/PVA/GLE organogel-based pressure sensor with current state-of-the-art flexible sensors

Material	Material Category	Device Configuration	Range (Kpa)	Sensitivity (kPa^−1^)	Dry resistance	Bio compatibility	Stability (cycles/Day)	Refs
PGEH	Hydrogel	Piezo–capacitive	1–10	1.25	– –	Yes	20,000	[41]
SF/Graphen	Aerogel	Capacitive	0.01–10	0.73	–	–	1000	[42]
PAM/PVA	Hydrogel	Piezo resistive	0–3.27	0.05	–	Yes	500	[43]
([VBIm][BF_4_])/([C_12_VIm][BF_4_])/([BMIm][BF_4_]	Ionogel	Piezo resistive	0.1–20	0.14	–	–	1000	[44]
PHEMA/PEGDA	Hydrogel	Piezo–resistive	0–2	0.24			–	[45]
SWCNT/ Alginate	Hydrogel	Piezo–resistive	0–0.01	0.176	–	–	–	[40]
Mxene/PU	Gel	Piezo–resistive	6.5–150 150–612	0.308 0.0697	–	–	> 20 days	[46]
Mxene/CS/PVDF	Aerogel	Piezo–resistive	0–106 106–400	0.11 0.055	–	–	6000	[47]
oxCNTs/GA/AM/AA/NMAM	Hydrogel	Piezo–resistive	0–10 20–80	2.27 0.08	–	Yes	80	[39]
LBG/PVA/CNTs	Hydrogel	Piezo–resistive	0–0.1 1–10 10–100	20.5 2.28 0.24		Yes	1000	[48]
SF/CNT	Hydrogel	Piezo–resistive	< 0.5 3–10	0.3 0.03–0.07		Yes	> 7000	[38]
**CoN CNT/PVA/GLE**	**Organogel**	**Piezo–capacitive**	**0–20**	**5.75**	**98%**	**Yes**	** > 100** **30 days**	**This work**

PGEH: polyacrylamide/gelatin/EGaIn; SF: silk Fibroin; PAM: Poly Acrylamide, PVA: Polyvinyl alcohol; ([VBIm][BF_4_]: 1-vinyl-3- butylimidazolium tetrafluoroborate, ([C_12_VIm][BF_4_]): 1-dodecyl-3-vinylimidazolium tetrafluoroborate, ([BMIm][BF_4_]: 1-butyl-3-methylimidazolium tetrafluoroborate; PHEMA: Poly(hydroxyethyl)methacrylate), PEGDA: Poly(ethylene glycol) diacrylate; SWCNT: Single-wall Carbon Nanotubes; CS: Chitosen, PVDF: Polyvinylidene fluoride; oxCNTs: oxidized multi-walled carbon nanotubes, GA: Gum Arabic, AA: acrylic acid, NMAM: N-methylolacrylamide; LBG: Locust Beam

### Bio-Mechanical Motion Monitoring

After comprehensively evaluating the mechanical, electrical, self-healing, and anti-freezing properties, we next demonstrate the potential of the pressure sensor for real-time wearable applications by monitoring body movements such as finger bending, wrist motion, and throat activity. To demonstrate its potential for real-time wearable applications, the organogel sensor was first affixed to a volunteer’s index finger to monitor bending motion. Upon finger bending, a sharp decrease in  device response  was observed (Fig. 5e), which remained consistent across multiple cycles, indicating stable and repeatable performance. A magnified view of a single bending cycle, along with corresponding stages of motion, is shown in Fig. 5h. The sensor was then evaluated at different bending angles (30°, 60°, and 90°) yielding response intensities of approximately 26.9%, 36.3%, and 49.1% respectively, (Fig. 5f), with a clear correlation between response magnitude and bending angle (Fig. 5i). The signal promptly returned to baseline when the finger was straightened, reflecting excellent mechanical recovery and response fidelity. Further, the sensor was tested under sustained bending conditions, with the finger held at fixed angles for 5 s. Stable plateaus were observed in each case (Fig. 5g and magnified in Fig. 5j), confirming the sensor’s ability to distinguish between dynamic and static strain. The response of the sensor to finger bending, after one week of ambient storage has been displayed in Fig. S31. The response remained stable, supporting mechanical resilience in a practical context. To expand the scope, the sensor was mounted on the throat and wrist. On the throat, it captured subtle vibrations during speech; when the volunteer articulated the letters “NMSL,” distinct response patterns emerged (Fig. S32). Notably, these waveforms closely resembled those generated when the same letters were written on the organogel surface, albeit with a slightly improved signal-to-noise ratio. On the wrist, the sensor successfully recorded upward and downward movements, as illustrated in Fig. S33. Together, these results underscore the sensor’s ability to detect a wide range of biomechanical motions with high precision and repeatability. Its reliable performance across diverse use cases highlights its potential in rehabilitation monitoring, healthcare diagnostics, and sports science.

### Biocompatibility and Environmental Impact Assessment

To evaluate the antimicrobial efficacy of the synthesized organogels, the standard agar well diffusion method-widely employed in clinical microbiology, was utilized. Zones of inhibition (ZOI), representing bacterial growth suppression, were measured on Day 1, Day 2, and Day 3. The results show that the CoN CNT/PVA/GLE organogel exhibited clear antimicrobial activity over three days, while the pristine PVA/GLE organogel did not inhibit *E. coli* growth during the same period [49]. The performance of both positive and negative controls further validates the reliability and integrity of the experimental setup. Figure 5k presents the ZOI images obtained on Day 1 and Day 3 (72 h), alongside a bar graph quantifying the corresponding inhibition diameters for each time point (Fig. S34 and Table S5). To further evaluate biosafety, cytotoxicity of CoN CNT-the principal functional component was evaluated via an MTT assay using RAW264.7 macrophage cells [50, 51]. The results (Fig. 5l and Table S6) show that cell viability remained above acceptable thresholds at concentrations of 12.5 and 6.25 µg mL^−1^, with no statistically significant difference when compared to the LPS-treated group (Fig. 5l). Moreover, as the cobalt nanoparticles are encapsulated within CoN CNT and further immobilized within the crosslinked organogel matrix as discussed earlier in microstructure analysis, thereby substantially reducing the risk of leaching and minimizing direct exposure. The final concentration of CoN CNT used in the organogel formulation was 0.1 mg mL^−1^, which is well within the safe range based on the cytotoxicity findings.

To evaluate the environmental footprint of the developed organogels, both pristine (PVA/GLE) and CoN CNT/PVA/GLE samples were subjected to oxidative degradation. Identical samples (1 cm × 1 cm × 0.3 cm, ~ 0.3 g) were immersed in 10 mL hydrogen peroxide (H_2_O_2_). Complete degradation was observed for both variants (Fig. S36 and Movie S5), supporting the organogels potential for environmentally benign disposal. Additionally, CoN CNT was synthesized via a low-energy, solid state method, eliminating the need for hazardous gases and high-vacuum systems typical of CVD (chemical vapor deposition) processes. Furthermore, the CoN CNT was used at low concentration (0.1 mg mL^−1^) and remained immobilized within the crosslinked matrix, thereby reducing the potential for nanoparticle release. A comparison table discussing environmental implication of both the synthesis route has been provided in Table S7.

### Electrical Output Performance to Recognize English Alphabet

**Fig. 6:**
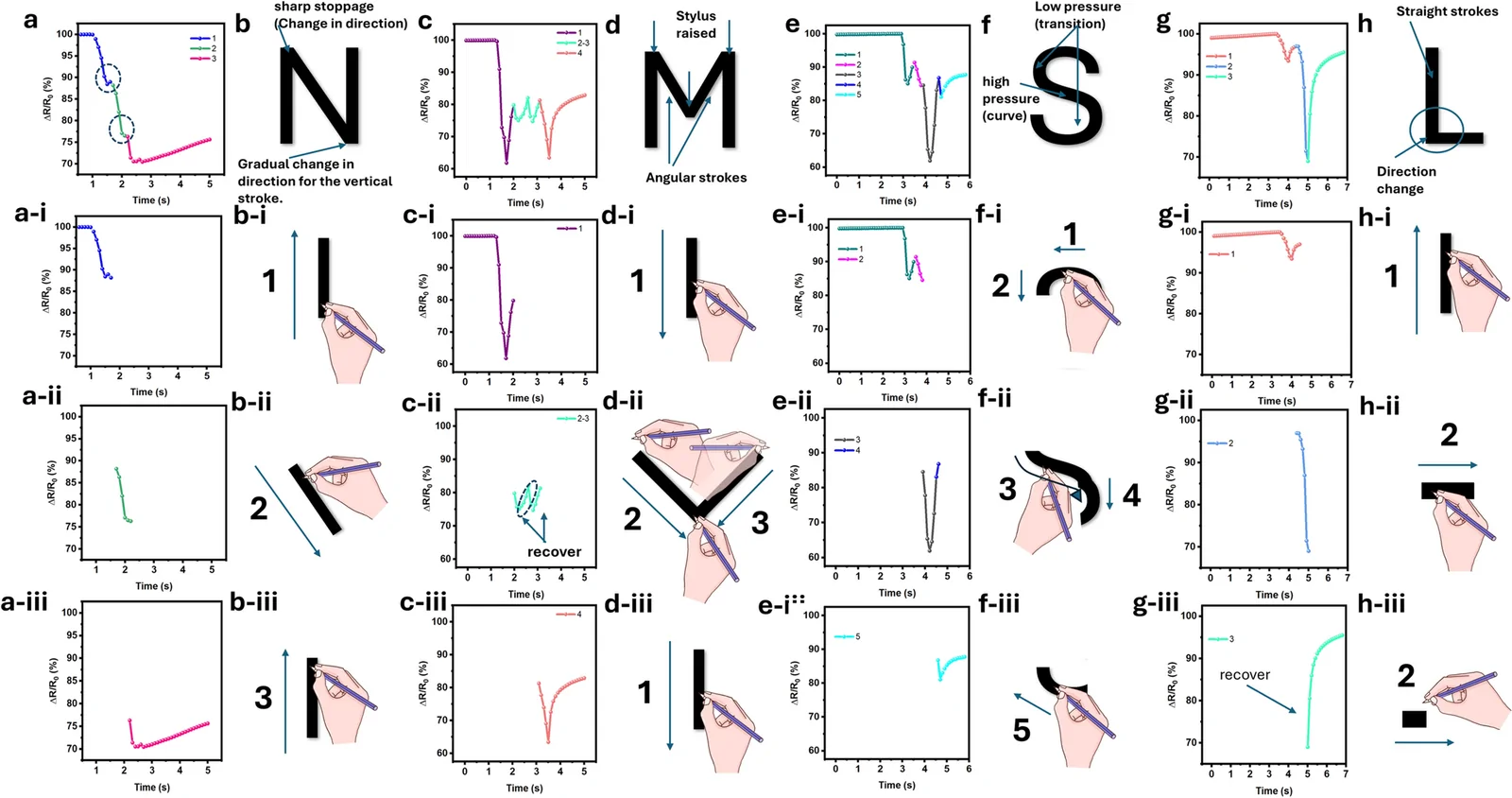
Demonstration of organogel sensor for character recognition and their respective resistive responses. **a** Resistive response for writing letter ‘N’, **ai–iii** detailing the response for each individual stroke during the writing of letter ‘N’. **b** Schematic representation of the writing process for letter ‘N’, **bi–iii** illustrating the sequence of strokes, stoppages, and directional movements. **c** Resistive response for writing letter ‘M’, **ci–iii** with a detailed breakdown of each stroke's contribution to the overall signal. **d** Schematic of the writing process for letter ‘M’, **di–iii** showing the direction and movement of each stroke. **e** Resistive response for letter ‘S’, **ei–iii** including a detailed analysis of each stroke and curve while writing. **f** Schematic illustration of writing letter ‘S’, **fi–iii** highlighting pressure variations and stroke sequences. **g** Resistive response for letter ‘L’, **gi–iii** with a breakdown of each stroke’s resistive behavior. **h** Writing mechanism of letter ‘L’, **hi–iii** showing the stroke sequence and movement patterns

Human handwriting is a dynamic process governed by multiple biomechanical and cognitive factors. At its core, letter formation involves not just continuous pen motion, but a complex interplay of pressure variations, pen-lifts, stoppages, and directional changes. Writing incorporates straight, curved, and angular strokes that require fine motor control. Each letter presents a characteristic motion pattern involving micro-pauses, directional shifts, and pressure modulation, making handwriting both unique and challenging to quantify. To effectively recognize these nuanced patterns, deep learning models were trained to accurately identify individual letters. For model training and validation, the letters L, M, N, and S were selected due to their diverse structural features: linear strokes (L), angular transitions (M and N), and continuous curves (S). This variety ensures coverage of the key geometric primitives in handwriting, enhancing the model’s ability to generalize across different writing styles [52]. The corresponding resistance response signatures and stroke sequences for each letter are shown in Fig. 6. Focusing on the letter ‘N’, its complete resistance response is presented in Fig. 6a, with associated writing strokes in Fig. 6b. The letter ‘N’ comprises three distinct strokes, each generating a unique resistance profile (Fig. 6a-i to a-iii), shaped by the pressure and motion dynamics. A sharp resistance changes marks the transition between the first and second strokes (circled in Fig. 6a), likely due to a brief stylus pause enabling a clean switch in direction. The transition from the second to third stroke is smoother, indicating a continuous motion with a less abrupt directional change. Importantly, the entire letter was written in one motion without lifting the stylus. The first stroke moves upward (Fig. 6b-i), followed by a sharp reversal (Fig. 6b-ii), and finally a smoother stroke forms the third line (Fig. 6b-iii). These transitions are clearly reflected in the resistance signal, offering valuable features for model training. For the letter ‘M’, four distinct strokes are required (Fig. 6d). The resistive response to the first downward stroke is shown in Fig. 6c-i, with the stylus lifted afterward, allowing the sensor to recover and resistance to rise slightly. The central angular structure is formed by two quick successive strokes, whose combined resistance response is shown in Fig. 6c-ii. Again, the stylus is briefly lifted after the second stroke (Fig. 6-dii), creating a marked recovery in resistance.

The fourth downward stroke completes the letter (Fig. 6-diii), with its signal captured in Fig. 6c-iii. These stroke-by-stroke resistive changes reflect stylus dynamics-including sequencing, pauses, and reapplication of force-providing a rich dataset for deep learning. In contrast, the letter ‘S’ is written in a single continuous motion without stylus lifting. It exhibits dynamic pressure modulation-with higher pressure at curved segments and lower pressure during transitions. Stroke patterns and corresponding resistance signals are shown in Fig. 6e-i/f-i, with the central curved motion detailed in Fig. 6e-ii/f-ii, and the lower arc in Fig. 6e-iii/f-iii. Subtle resistance variations result from continuous curvature and stylus speed changes. The letter ‘L’ comprises two strokes-a vertical downward followed by a horizontal extension-written without stylus lift. These strokes and their resistance responses are presented in Fig. 6g, h. Despite its simplicity, the orthogonal stroke transition produces a clear and repeatable resistance signature.

Sensor repeatability and robustness are demonstrated in Fig. S37, where resistance responses for 10 consecutive writing cycles of each letter (N’, ‘M’, ‘S’, and ‘L’) show high consistency. Letters like ‘N’ and ‘M’ combine straight lines and sharp turns requiring stylus lifting, while ‘S’ presents pressure-varying curved motions. Even the simple ‘L’ encapsulates distinct directional transitions, critical for character distinction. Beyond these examples, the CoN CNT/PVA/GLE organogel sensor was used to recognize all 26 English letters (A-Z) and numeric digits (0–9), each yielding distinct resistive signatures (Figs. S38 and S39). Each character comprises different combinations of strokes and curvatures, altering the local strain distribution within the organogel matrix and consequently the measured resistance. Pause-start behavior and curved movements generate unique resistance patterns for each letter. The viscoelastic nature of the CoN CNT/PVA/GLE organogel introduces path dependent signal variations. Residual strain from one stroke may influence the subsequent one, particularly in fast or continuous writing. As a result, each letter exhibits a distinctive electrical signature, enabling reliable and precise recognition.

### Machine Learning-Assisted Alphabet Recognition

To enable machine learning assisted character recognition, each letter (L, M, N, and S) was written 150 times on the sensor, generating a comprehensive dataset. Continuous resistance signals were segmented using a sliding window (window size = 50, step size = 10), resulting in 1507, 1606, 1486, and 1725 labeled time-series samples for the letters L, M, N, and S respectively, as summarized in Table TS8. These samples were subsequently stratified into training and testing sets to ensure balanced class representation during model evaluation. To classify the resistance signals corresponding to each handwritten letter, we employed various machine learning algorithms. Performance was assessed using accuracy, precision, recall, and F1-score (Table S9). Among all models, the XGBoost classifier achieved the highest overall accuracy and F1-score, while a stacked ensemble model combining CNN-LSTM and XGBoost demonstrated robust and consistent performance with minimal misclassifications. These errors primarily arose from similarities in signal patterns between letters or variations in motion dynamics during writing. Each sample in the dataset was preprocessed by removing missing values and extracting windowed features, including statistical descriptors such as mean, standard deviation, maximum, minimum, and range for both resistance and time. Features were standardized using StandardScaler, and class labels were encoded with LabelEncoder. The CNN-LSTM model was trained using the Adam optimizer (learning rate = 0.001), over 200 epochs with a batch size of 32.

The XGBoost classifier was configured with 100 estimators and a learning rate of 0.1. To improve generalization, we used logistic regression as the meta-classifier for the stacking ensemble to combine predictions from CNN-LSTM and XGBoost. To evaluate model robustness under realistic variability, participants were instructed to alter writing speed and applied pressure deliberately. This introduced significant fluctuations in the resistance signals (Fig. S40), simulating real-world usage conditions. Under these perturbed conditions, model performance declined: CNN-LSTM accuracy dropped from 86.56% to 83.83%, XGBoost from 98.26% to 94.88%, and the stacking model from 98.18% to 95.33%. These findings confirm that variations in writing dynamics, such as frequency and pressure, substantially affect signal profiles and pose a challenge for accurate recognition.

## Conclusions

This work presents a self-healing, biocompatible organogel pressure sensor engineered by incorporating CoN CNT into a PVA-GLE matrix, offering a compelling platform for next-generation wearable electronics. The device exhibits high sensitivity (5.75 kPa^−1^), excellent linearity, and sustained performance over one month in the 0–20 kPa range. Crucially, the sensor demonstrates robust environmental stability, retaining functionality across a wide humidity (40%–80% RH and temperature (−20 to 45 °C) window. Electrical stability under even harsher conditions (40–95% RH, −20 to 60 °C) was validated via electrochemical impedance spectroscopy. Additionally, an intelligent human–machine interface system, leveraging deep learning for English letter recognition, achieved an impressive 98% accuracy. Multiple machine learning models-including XGBoost, 1D CNNs, and LSTM networks-were employed to classify time-series data, with a stacking classifier further enhancing performance. The organogel's ability to monitor finger bending, wrist movements, and throat vibrations positions it as a transformative tool for healthcare, rehabilitation, and HMI applications, seamlessly integrating advanced materials with AI to enable a more intuitive, responsive future.

## Supplementary Information

Below is the link to the electronic supplementary material.

Supplementary file1 (MP4 1607 KB)

Supplementary file2 (MP4 577 KB)

Supplementary file3 (MP4 413 KB)

Supplementary file4 (MP4 382 KB)

Supplementary file5 (MP4 2306 KB)

Supplementary file6 (DOCX 28818 KB)
