# Supplementary material for: Deep Learning-Assisted Organogel Pressure Sensor for Alphabet Recognition and Bio-Mechanical Motion Monitoring
Source: Nanomicro Lett. 2025 Sep 8;18:63. doi: 10.1007/s40820-025-01912-z (PMC12420570; doi:10.1007/s40820-025-01912-z)
Supplement: Supplementary file 6 — Supplementary file6 (DOCX 28818 KB) [file 40820_2025_1912_MOESM6_ESM.docx]

Supporting Information for

**Deep Learning Assisted Organogel Pressure Sensor for Alphabet Recognition and Bio-Mechanical Motion Monitoring**

Kusum Sharma^1#^, Kousik Bhunia^1#^, Subhajit Chatterjee^2^, Muthukumar Perumalsamy^1^, Anandhan Ayyappan Saj^1^, Theophilus Bhatti^6^, Yung-Cheol Byun^3^, Sang-Jae Kim^1,4,5,7, *^

^1^ Nanomaterials & System Lab, Major of Mechatronics Engineering, Faculty of Applied Energy System, Jeju National University, Jeju 63243, Republic of Korea

^2^ Department of Computer Engineering, Jeju National University, Jeju-si, 63243, Republic of Korea

^3^ Department of Computer Engineering, Major of Electronic Engineering, Jeju National University, Institute of Information Science & Technology, Jeju 63243, Republic of Korea

^4^ Nanomaterials & System Lab, Major of Mechanical System Engineering, College of Engineering, Jeju National University, Jeju 63243, Republic of Korea

^5^ Research Institute of Energy New Industry (RINEI), Jeju National University, Jeju 63243, Republic of Korea

^6^ Interdisciplinary Graduate Program in Advanced Convergence Technology & Science, Jeju National University, Republic of Korea

^7^ Green Hydrogen Glocal Leading Research Center (gH_2_-RC), Jeju National University, Jeju 63243, Republic of Korea

**^#^** Kusum Sharma and Kousik Bhunia contributed equally to this article.

*Corresponding author. E-mail: [kimsangj@jejunu.ac.kr](mailto:kimsangj@jejunu.ac.kr) (Sang-Jae Kim)

**S1 Synthesis of CoN CNT**

In a typical synthesis method, 1 mmol of Co(NO_3_)_3_.6H_2_O is dissolved in 50 mL absolute ethanol. Subsequently, 10 g of melamine is added under continuous stirring. The mixture was placed over a preheated hotplate at 80 °C to evaporate the solvent. The dried mixture was collected in a semi-closed crucible and annealed in a muffle furnace at 550 °C for 2 h at a heating rate of 5 °C min^-1^. Subsequently, the sample was collected and crushed to powder. Obtained powder is placed in a tubular furnace and heated under an argon atmosphere for 2 h at 850 °C with a heating rate of 5°C min^-1^. The synthesized CoN CNT has been directly used for the organogel preparation.

**S2 Characterization**

The surface morphology and cross-sectional parts were analyzed using a Field Emission Scanning Electron Microscope (FE-SEM, TESCAN MIRA3). The high-resolution transmission electron micrograph (HR-TEM) for the CoN CNT was carried out on an HR-TEM, at Korea basic science institute (KBSI), Busan. The X-ray diffractions (XRD) were carried out using Malvern PANalytical, Empyrean, with a Cu K-alpha source. The vibrational modes and functional groups were analyzed using Fourier Transform Infrared spectroscopy (Bruker, Alpha II). X-Ray Spectroscopy measurements were performed (XPS, Nexa, ThermoFisher Scientific, UK) with x-ray source Al Ka (1486.6 eV) from Busan Center Korea Basic Science Institute (KBSI), South Korea. The pressure-dependent capacitive measurements were performed using an impedance analyzer (HIOKI 3570). The EIS measurements were performed on VIONIC in the frequency range from 1MHz to 100 mHz with a 10 mV test signal. The resistive response measurements were systematically measured using a semiconductor device analyzer (Agilent B1500) and electrometer (Keithley 6514).

**S3 Deep Learning-Based Models and Validation**

**3.1 1D-CNN Model**

One-dimensional convolutional neural networks (1D-CNN) are very suitable for extracting spatial patterns from time series data. This model used a single convolutional layer with 64 filters and a kernel size of 2. The convolutional layer was followed by a max pooling layer to reduce dimensionality. The 1D-CNN performed well on its own but was further improved when combined with an LSTM layer to capture the temporal dependence of the data.

**3.2 LSTM Model**

Given that time-series tasks require capturing temporal patterns, LSTMs are very powerful as they hold entity memory units. LSTM involves input, forget and output gates which control the information that flows. The LSTM has memory cells that can help the model capture dependencies in time-series data making it particularly valuable for identifying sequential patterns within organogel sensor signals. In these experiments, the LSTM model with two stacked LSTM layers of 64 hidden units had been used to capture long-term dependency in the signal patterns across letters. Dropout was applied through the LSTM layers for regularization against overfitting. Diagram of the LSTM network architecture as shown in Scheme S1.


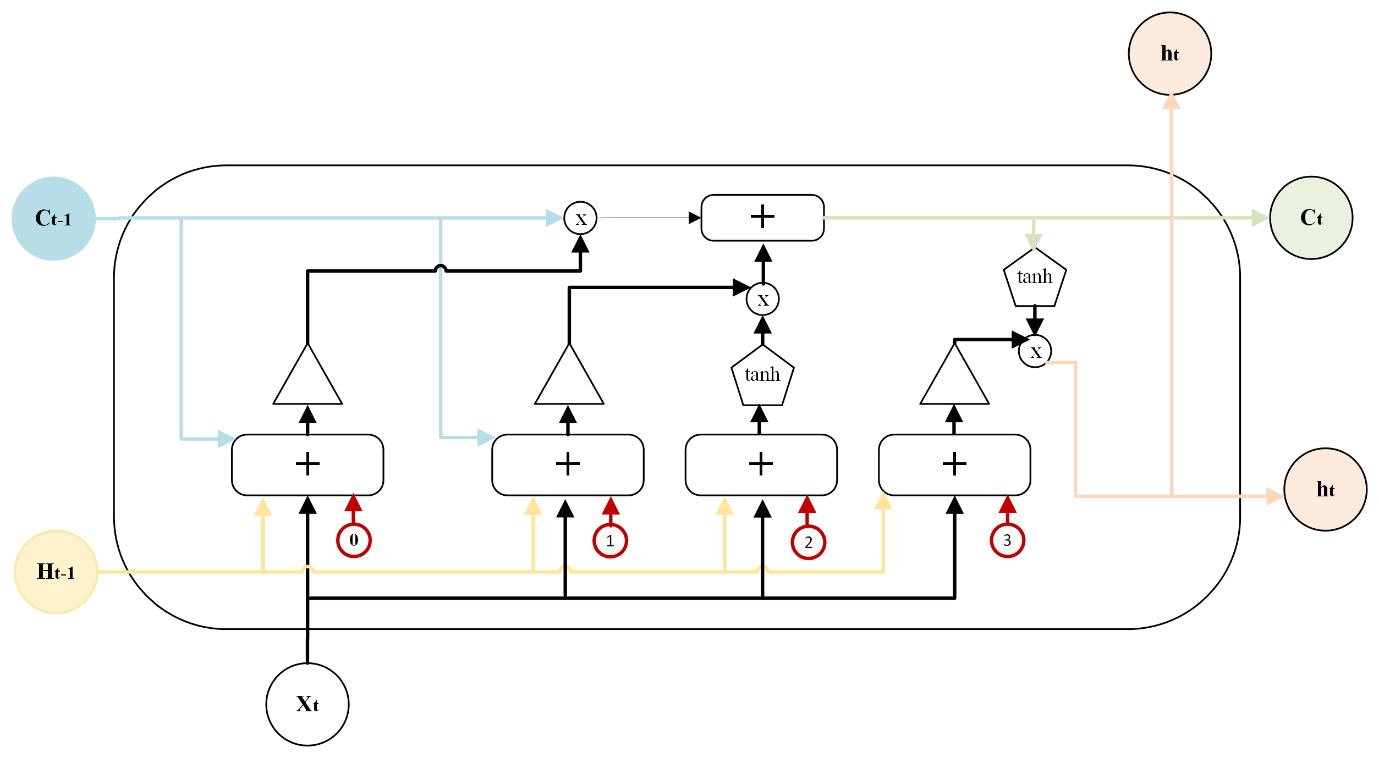
**Scheme S1** Network architecture of LSTM

**3.3 XGBoost Model**

XGBoost leverages gradient boosting to combine multiple decision trees, where each subsequent tree corrects the mistakes of the previous one. XGBoost is well known for its efficiency and accuracy in structured data tasks. It was configured with 100 estimators and a learning rate of 0.1. The XGBoost model performed remarkably well in this task, significantly boosting the accuracy compared to deep learning models alone. The architecture of the XGBoost classifier used in this study is shown in Scheme S2. This method is particularly efficient in handling structured time-series data, allowing for robust classification of resistance signals from the organogel sensor. The model's ability to optimize both accuracy and speed makes it a valuable tool in classifying the distinct signal patterns associated with each letter.


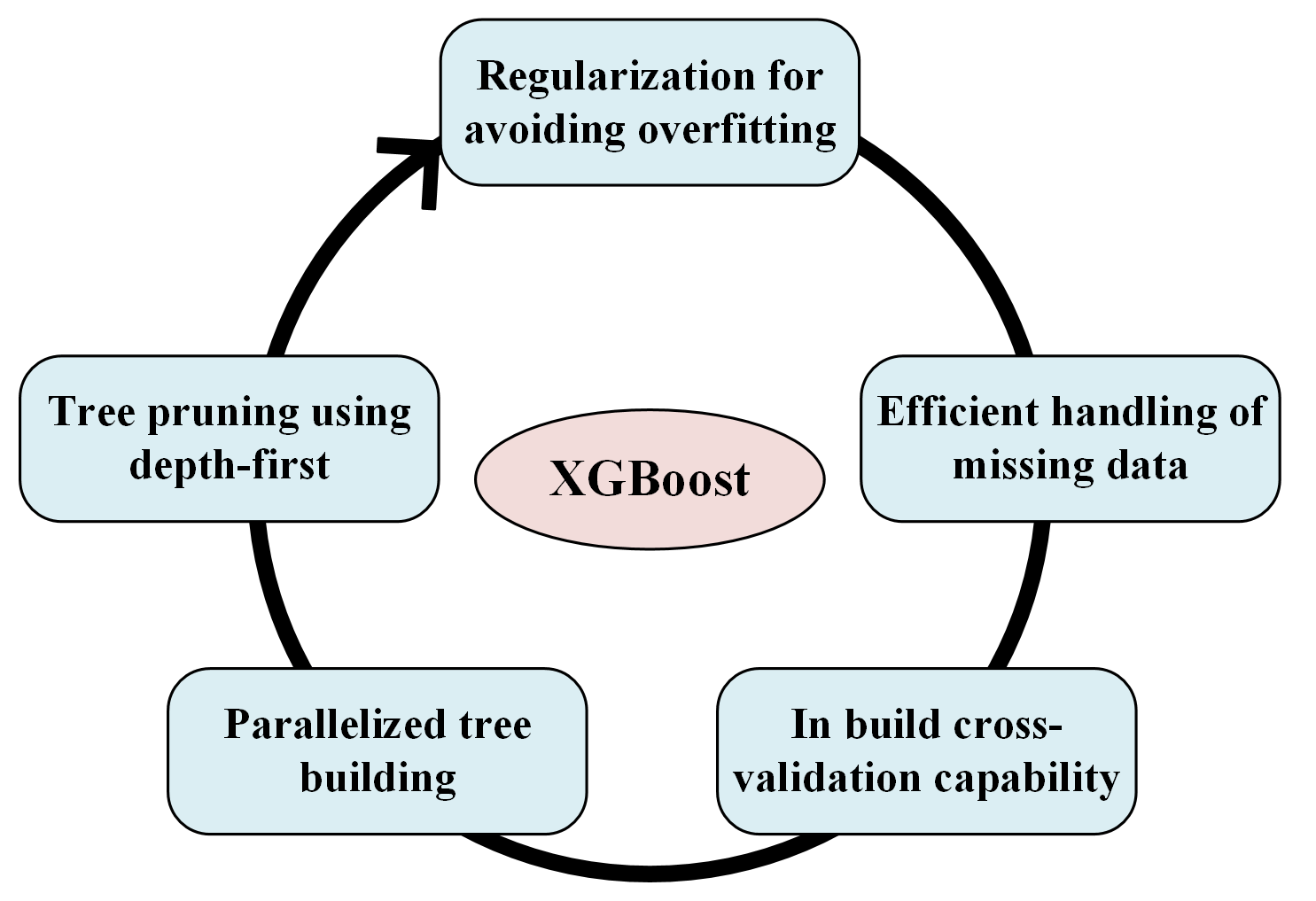


**Scheme** **S2** Architecture of XGBoost classifier

**3.4 Stacking Classifier**

Finally, we combined the CNN-LSTM and XGBoost models in a stacking classifier. The stacking approach employed a logistic regression model as the meta-learner to integrate the predictions from both base models. This ensemble strategy leverages the complementary strengths of deep temporal modeling (via CNN-LSTM) and gradient boosting (via XGBoost), thereby enhancing the overall classification performance.


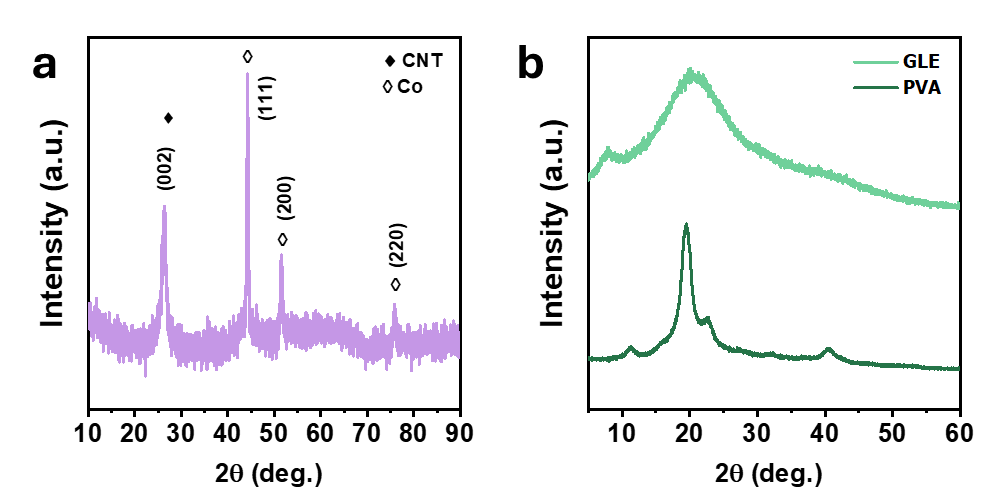


**Fig. S1** Powder XRD pattern of **a** CoN CNT, and **b** pure PVA and gelatin powder.

Pristine PVA powder exhibits sharp reflections at 19.5° and 22.7°, representing the (101) and (101*) planes of the monoclinic unit cell of PVA and a small peak at 40.5°, exhibiting the semicrystalline nature of PVA resulting from the strong intermolecular hydrogen bonding. Gelatin powder displays a halo at 20° and a sharp peak at 8°, corresponding to the triple helix molecule of gelatin.


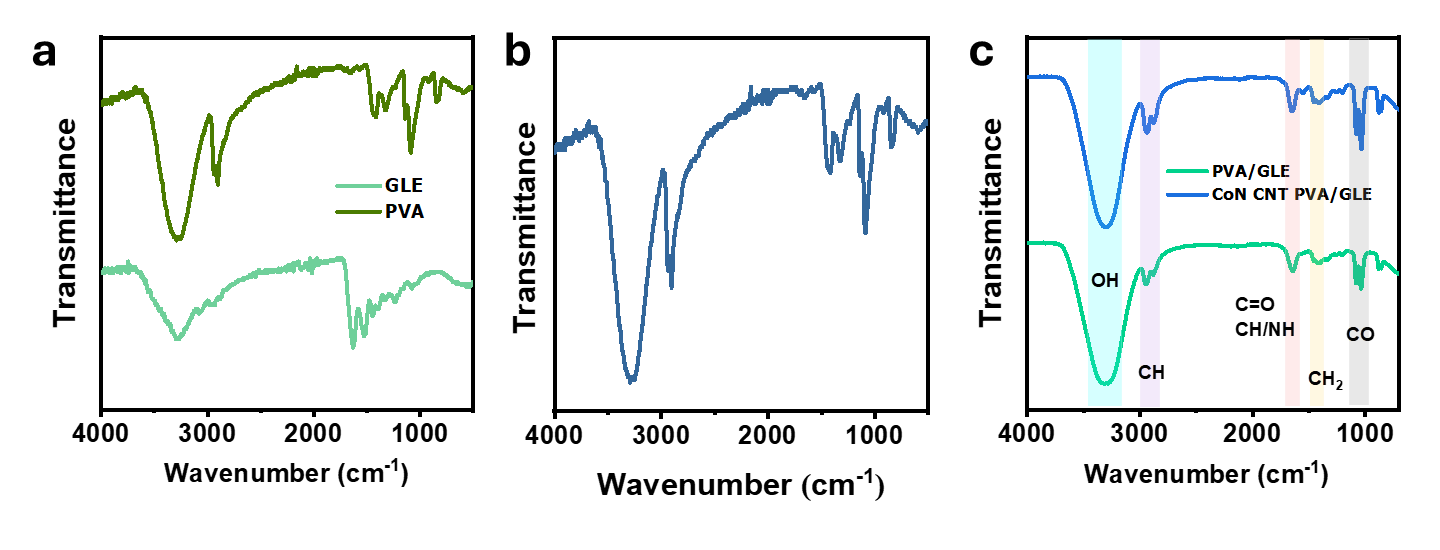


**Fig. S2** FTIR spectra of **a** pure PVA, and gelatin powder, **b** Ethylene glycol (EG)

All the characteristics peak 3278 cm^-1^, 2932/2901 cm^-1^, 1411 cm^-1^, 1316 cm^-1^, 1133 cm^-1^, 916 cm^-1^, and 853 cm^-1^ respectively represent the OH stretching, CH stretching, CH_2_ bending, OH rocking with CH wagging, C-O stretching, CH_3_ rocking and C-C stretching.


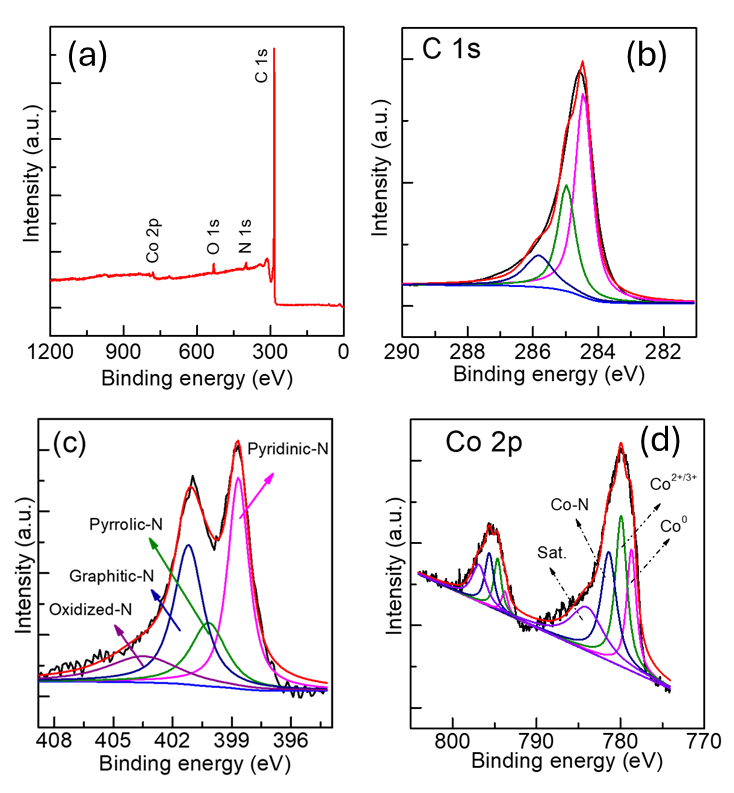


**Fig. S3 a** Survey spectrum, high resolution XPS of **b** C1s, **c** N1s, and **d** Co 2p of pristine CoN CNT


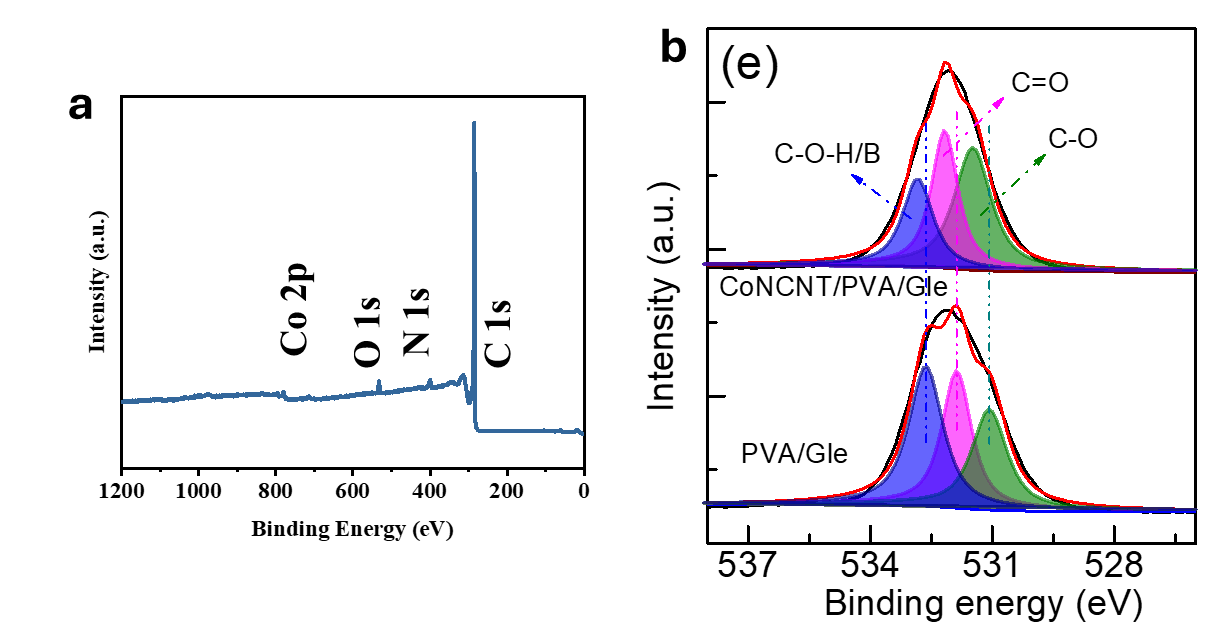


**Fig.** **S4** High resolution spectra of O 1s of CoN CNT/PVA/GLE and PVA/GLE

**
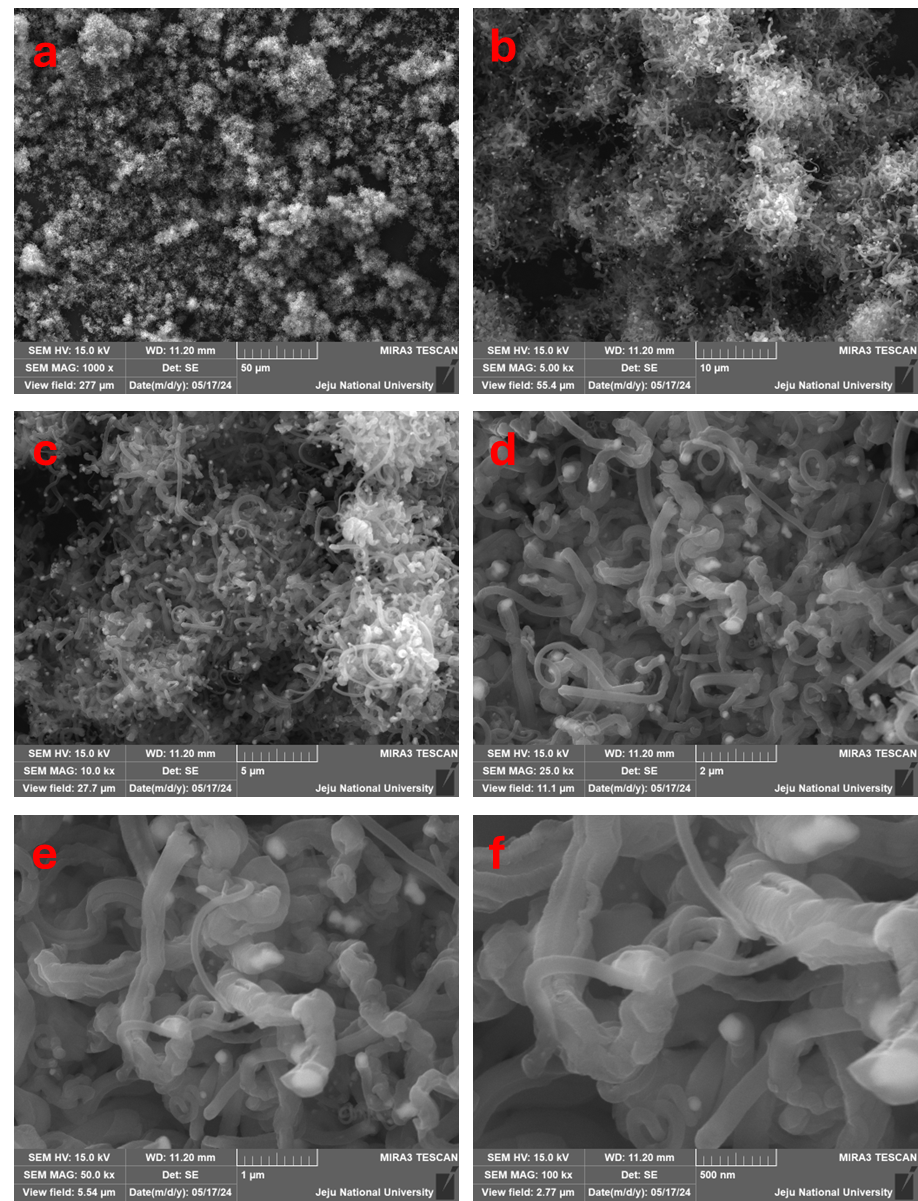
**

**Fig. S5** FESEM images of the synthesized CoN CNT at different magnifications


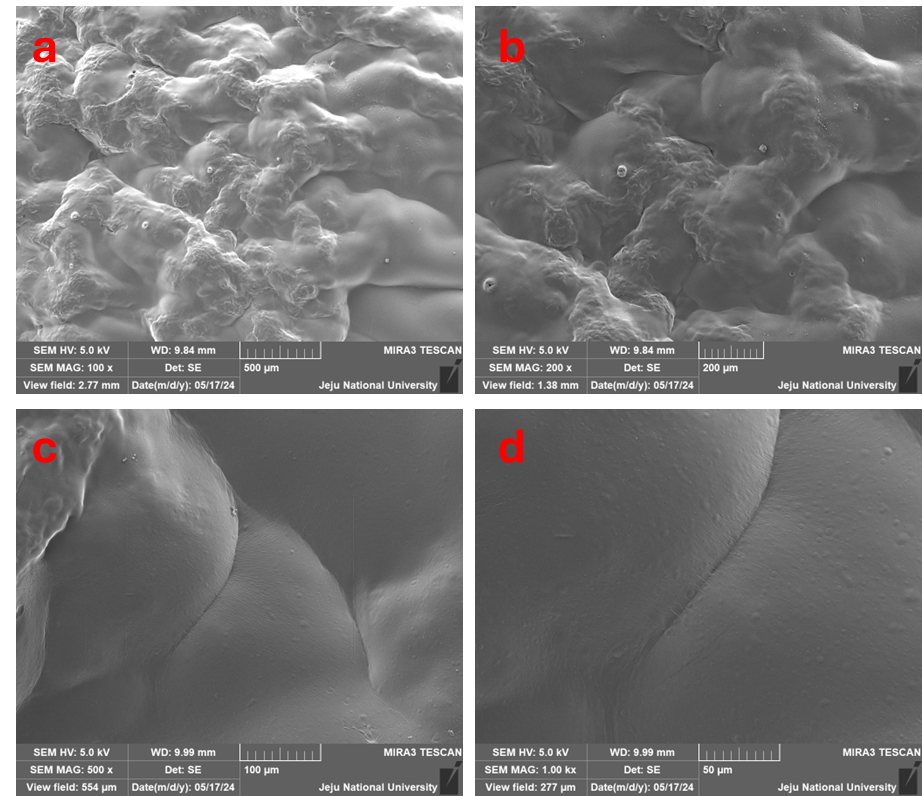


**Fig.** **S6** FESEM image showing the surface morphology of PVA/GLE organogel at various magnifications


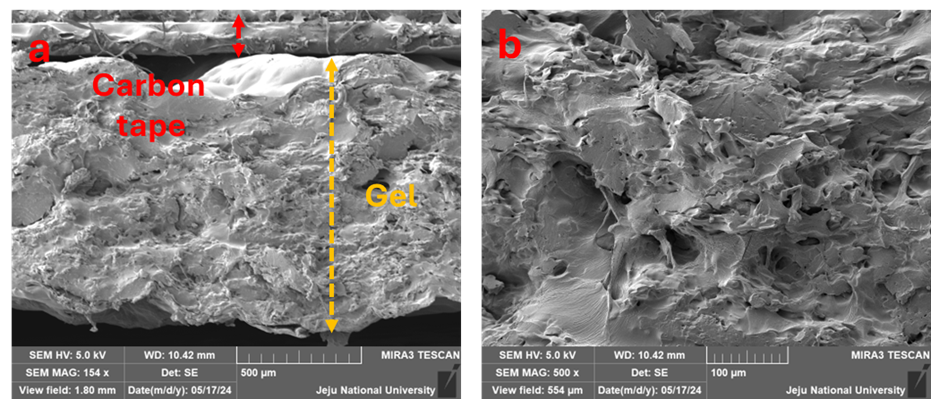


**Fig. S7 a-b** Cross-sectional view of PVA/GLE matrix at various magnification


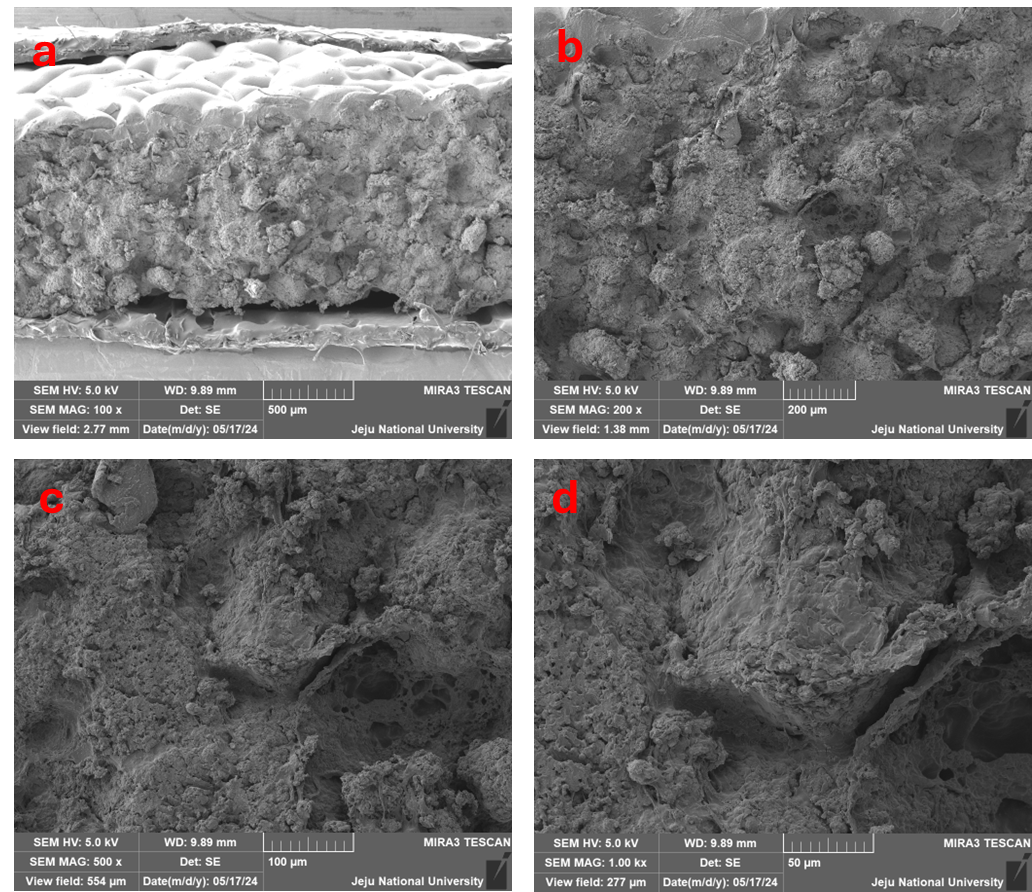


**Fig. S8 a-d** Cross-sectional view of CoN CNT/PVA/GLE matrix at various magnification

**Table S1** Summarized anti-fatigue results.

| No. of Cycle  (n) | ED (Original)  kJ/m^3^ | ED (10 min)  kJ/m^3^ | RED (Original)  % | RED (10 min)  % |
| --- | --- | --- | --- | --- |
| 1 | 8.93 | 3.63 | 100 | 40.6495 |
| 2 | 2.79 | 1.62 | 31.243 | 18.1411 |
| 3 | 2.2 | 1.37 | 24.6361 | 15.3416 |
| 4 | 1.96 | 1.3 | 21.9485 | 14.5577 |
| 5 | 1.81 | 1.19 | 20.2688 | 13.3259 |
| 6 | 1.73 | 1.16 | 19.3729 | 12.9899 |
| 7 | 1.65 | 1.15 | 18.477 | 12.8779 |
| 8 | 1.6 | 1.14 | 17.9171 | 12.766 |
| 9 | 1.54 | 1.13 | 17.2452 | 12.654 |
| 10 | 1.49 | 1.1 | 16.6853 | 12.318 |

#ED : Energy dissipation & RED: Relative Energy Dissipation


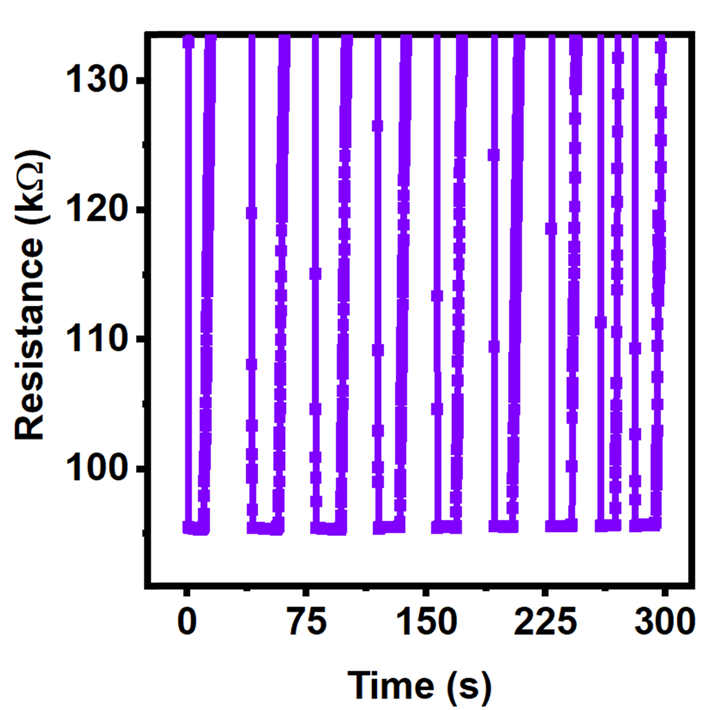


**Fig. S9** Magnified view of resistance variation upon cut and attach, exhibiting the base resistance value while healing process


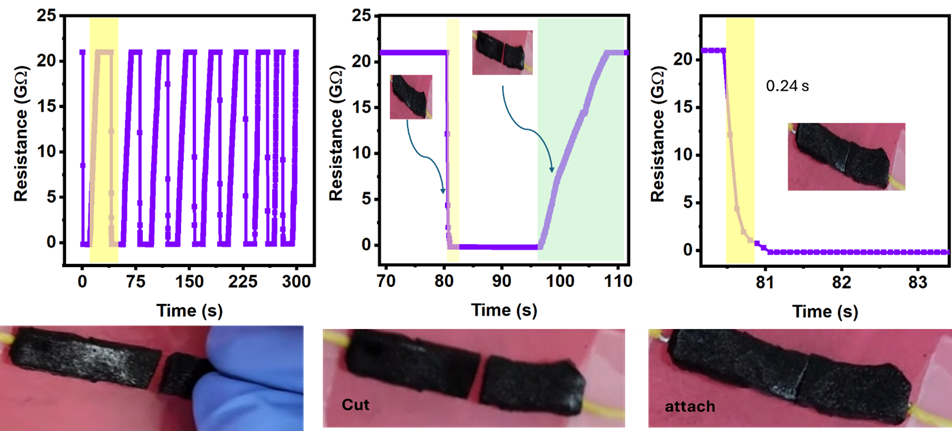


**Fig. S10** The magnified view of self-healing process exhibiting the time to recover electrically along with digital images


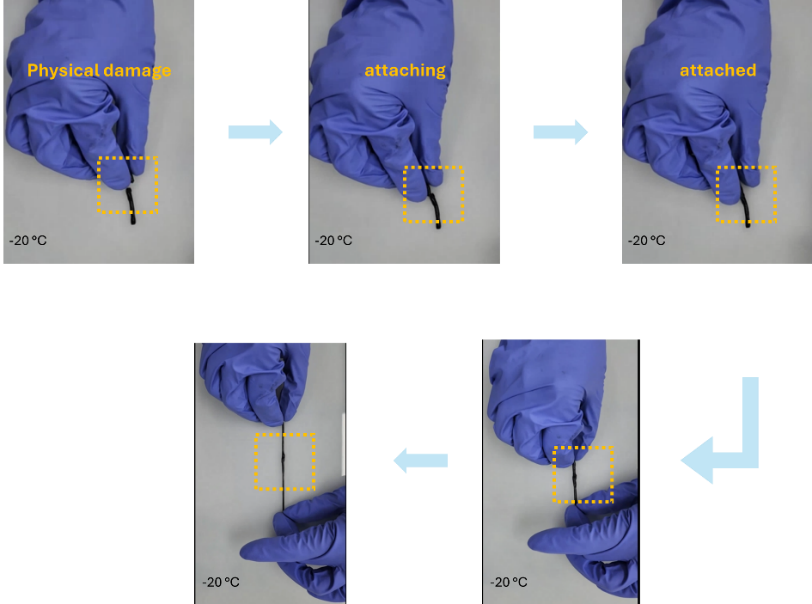


**Fig. S11** Digital images show the instantons self-healing process for the gel stored under sub-zero conditions


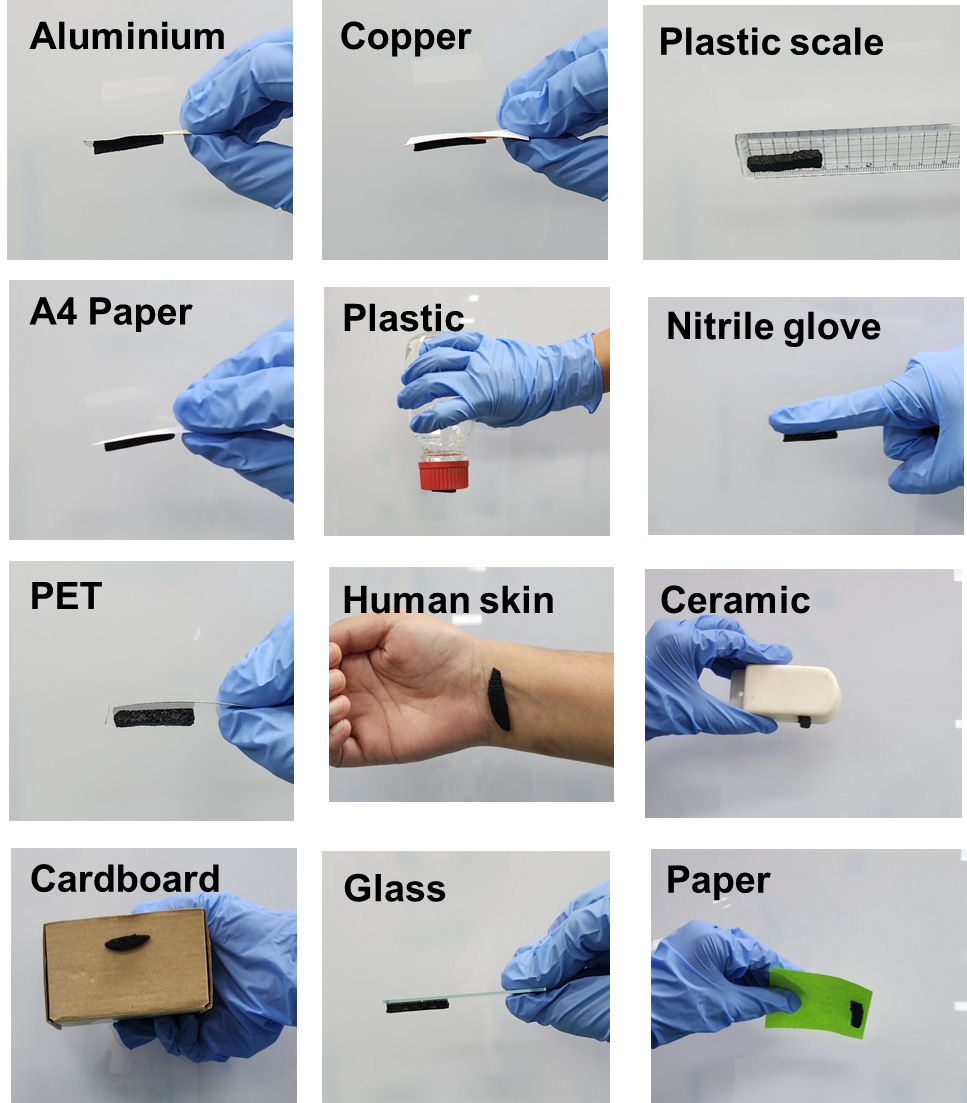


**Fig. S12** Adhesion test of CoN CNT/PVA/GLE organogel on various materials at various angles


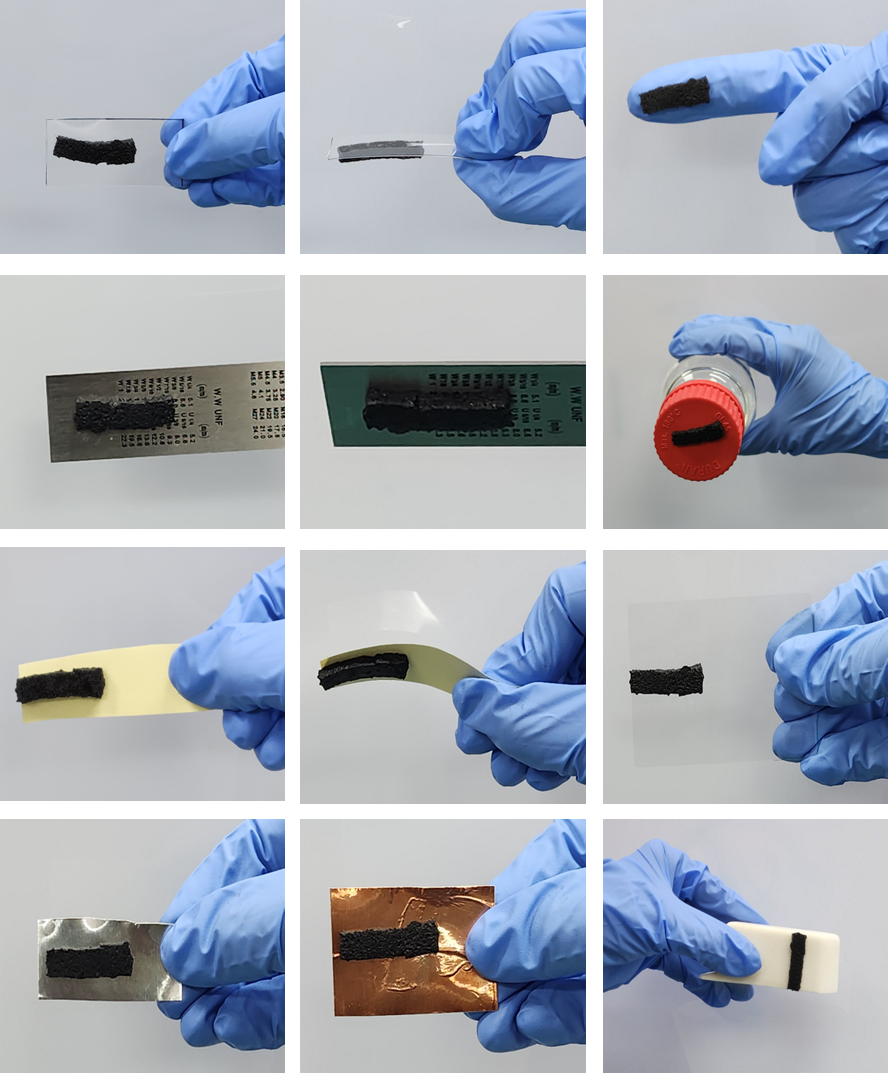


**Fig. S13** Adhesion test of CoN CNT/PVA/GLE organogel on various materials at various angles


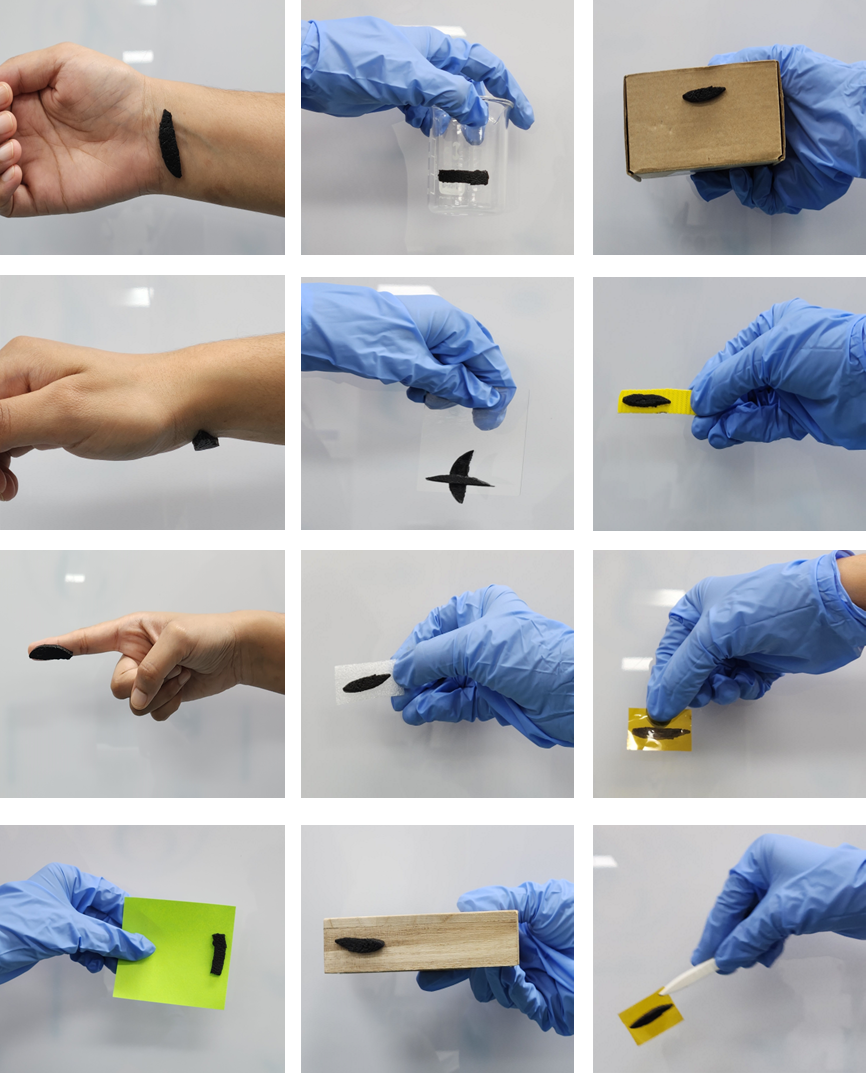


**Fig. S14** Adhesion test of CoN CNT/PVA/GLE organogel on various materials at various angles


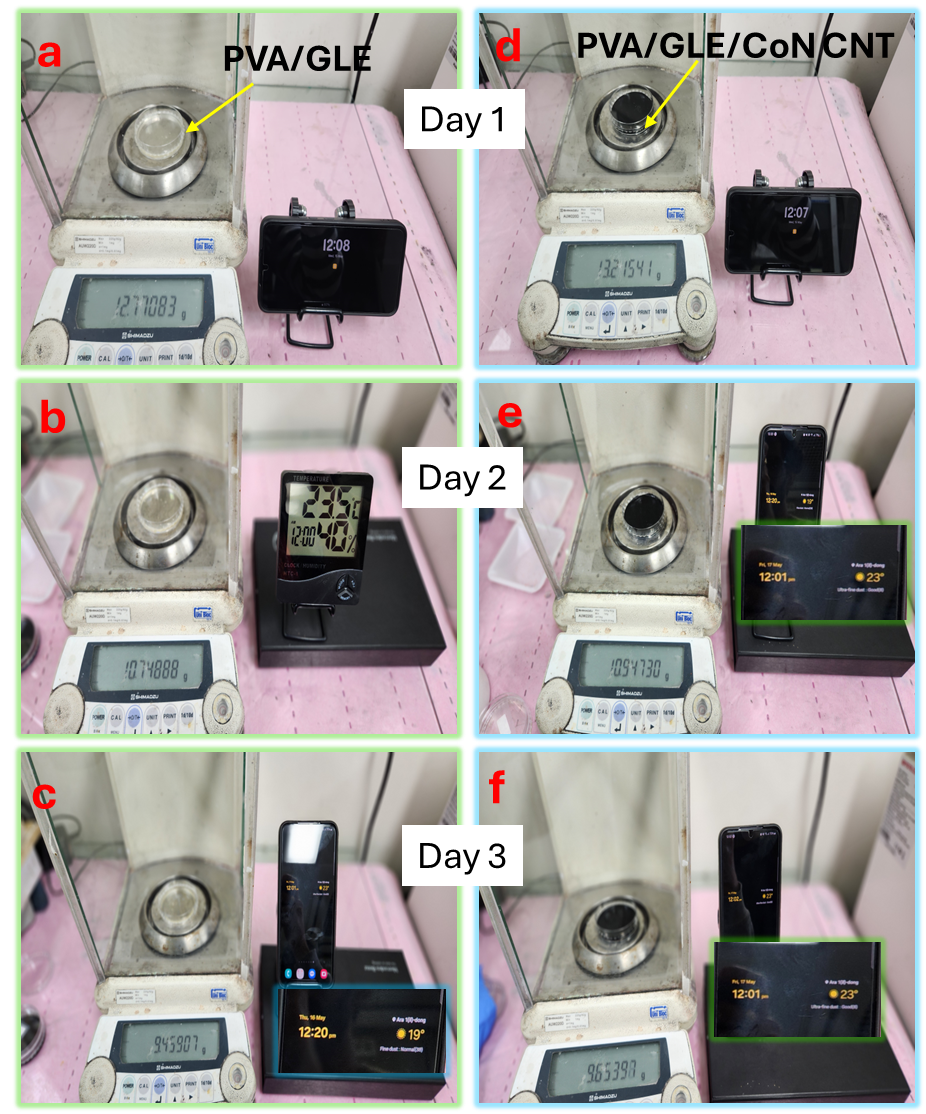


**Fig. S15** Weight retention analysis of **a-c** PVA/GLE, and **d-f** CoN CNT/PVA/GLE organogel under ambient conditions over three days. Images show the mass measurements on Day 1, Day 2, and Day 3


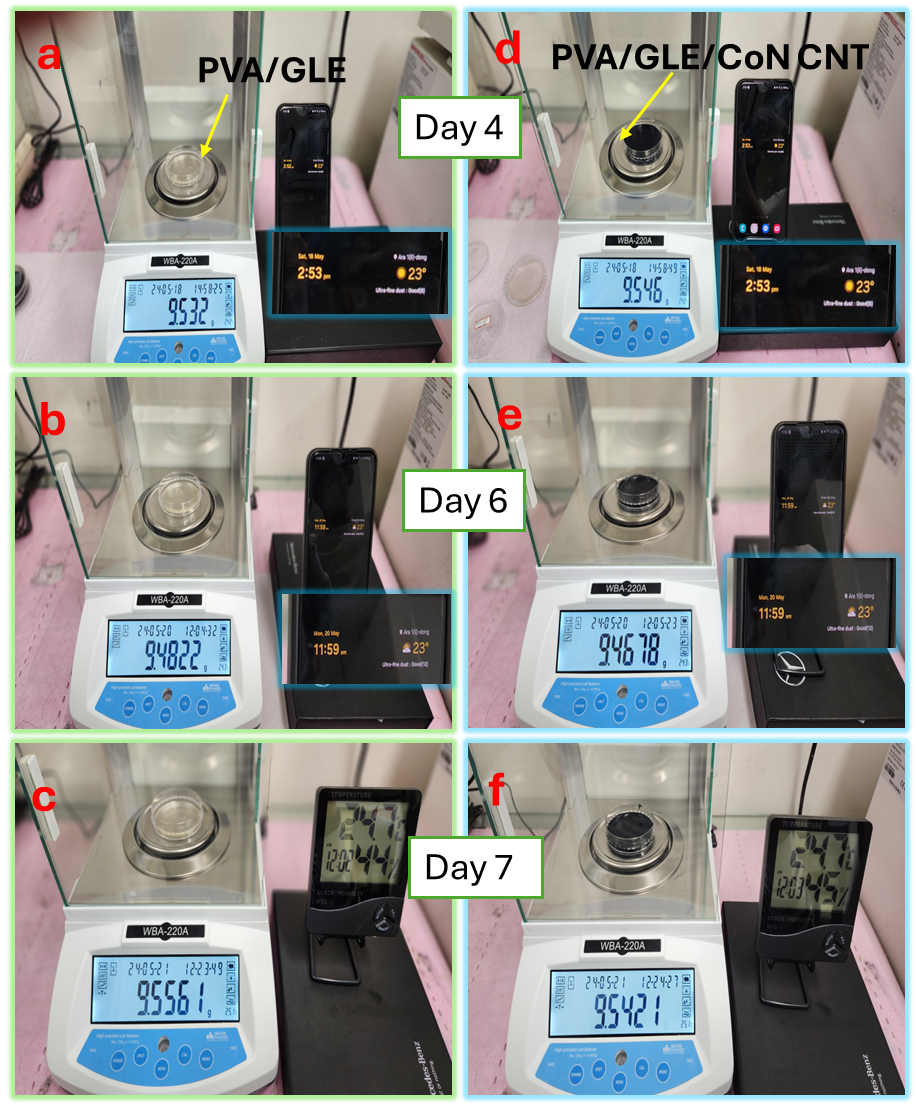


**Fig. S16** Weight retention analysis of **a-c** PVA/GLE, and **d-f** CoN CNT/PVA/GLE organogel under ambient conditions over three days. Images show the mass measurements on Day 4, Day 5, and Day 7


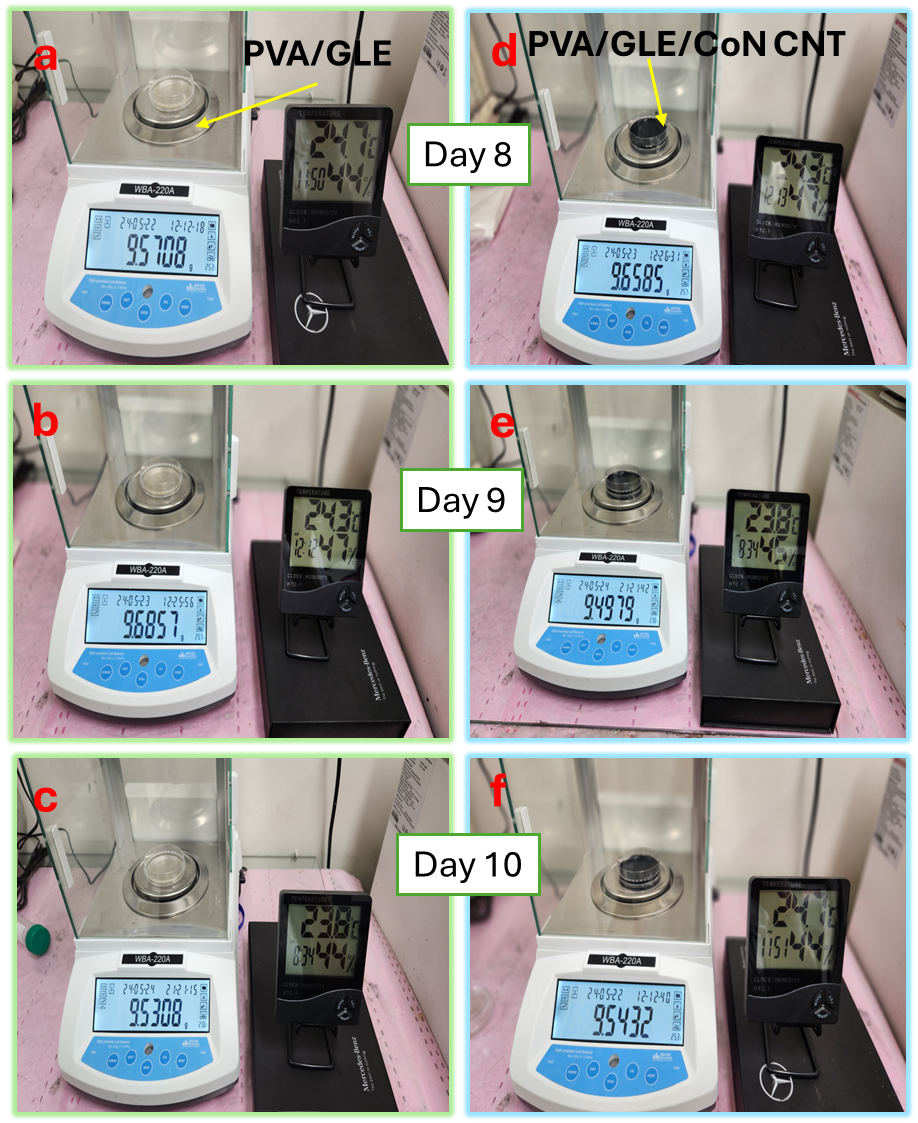


**Fig. S17** Weight retention analysis of **a-c** PVA/GLE, and **d-f** CoN CNT/PVA/GLE organogel under ambient conditions over three days. Images show the mass measurements on Day 8, Day 9, and Day 10


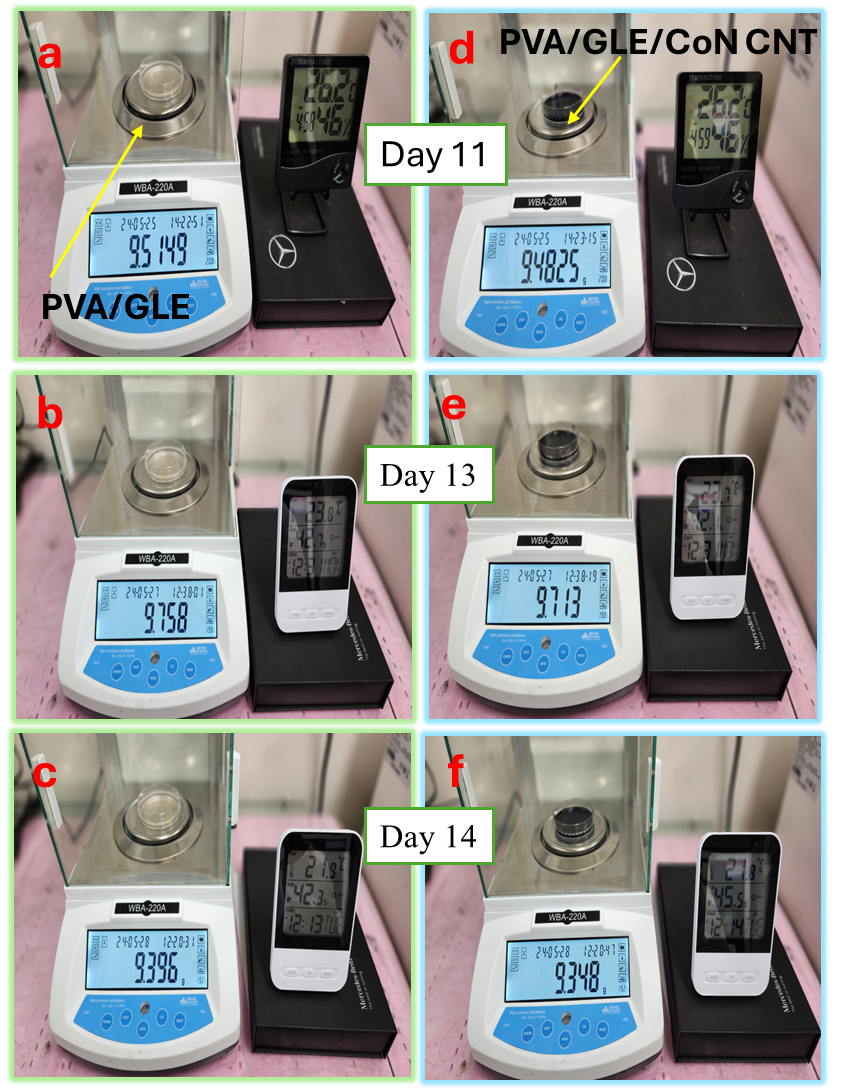


**Fig. S18** Weight retention analysis of **a-c** PVA/GLE, and **d-f** CoN CNT/PVA/GLE organogel under ambient conditions over three days. Images show the mass measurements on Day 11, Day 13, and Day 14


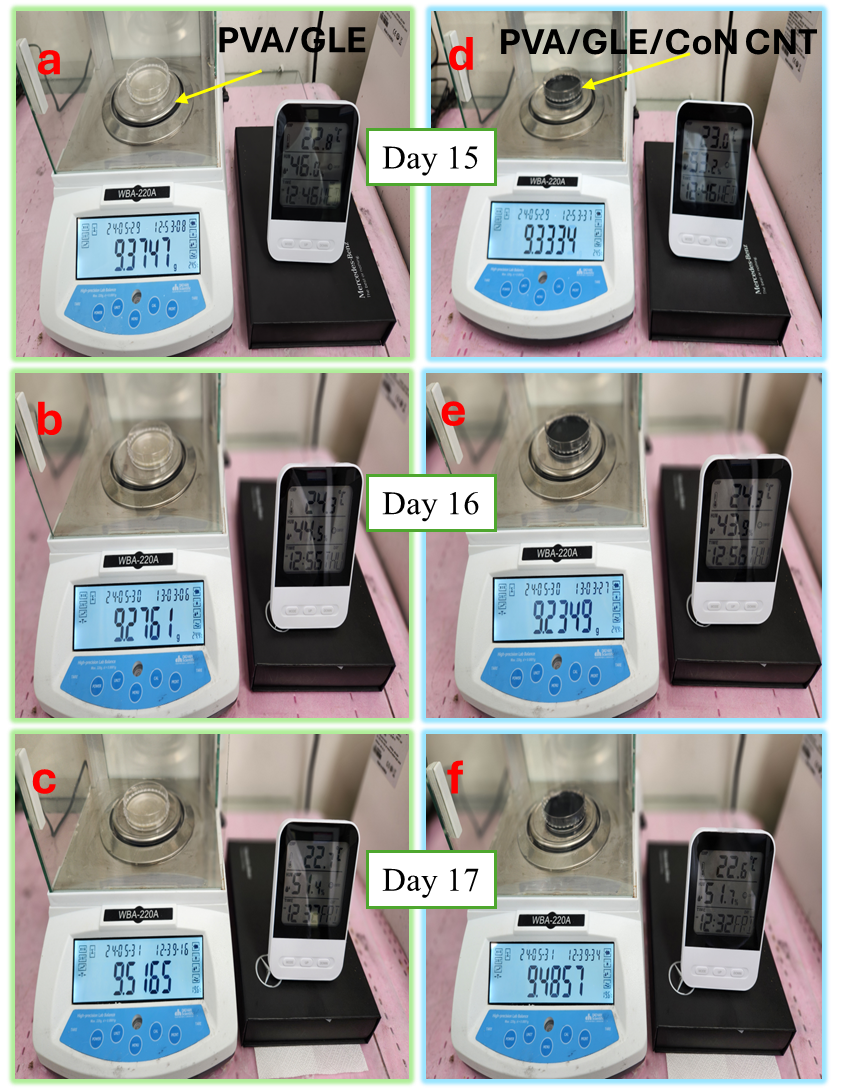


**Fig. S19** Weight retention analysis of **a-c** PVA/GLE, and **d-f** CoN CNT/PVA/GLE organogel under ambient conditions over three days. Images show the mass measurements on Day 15, Day 16, and day 17


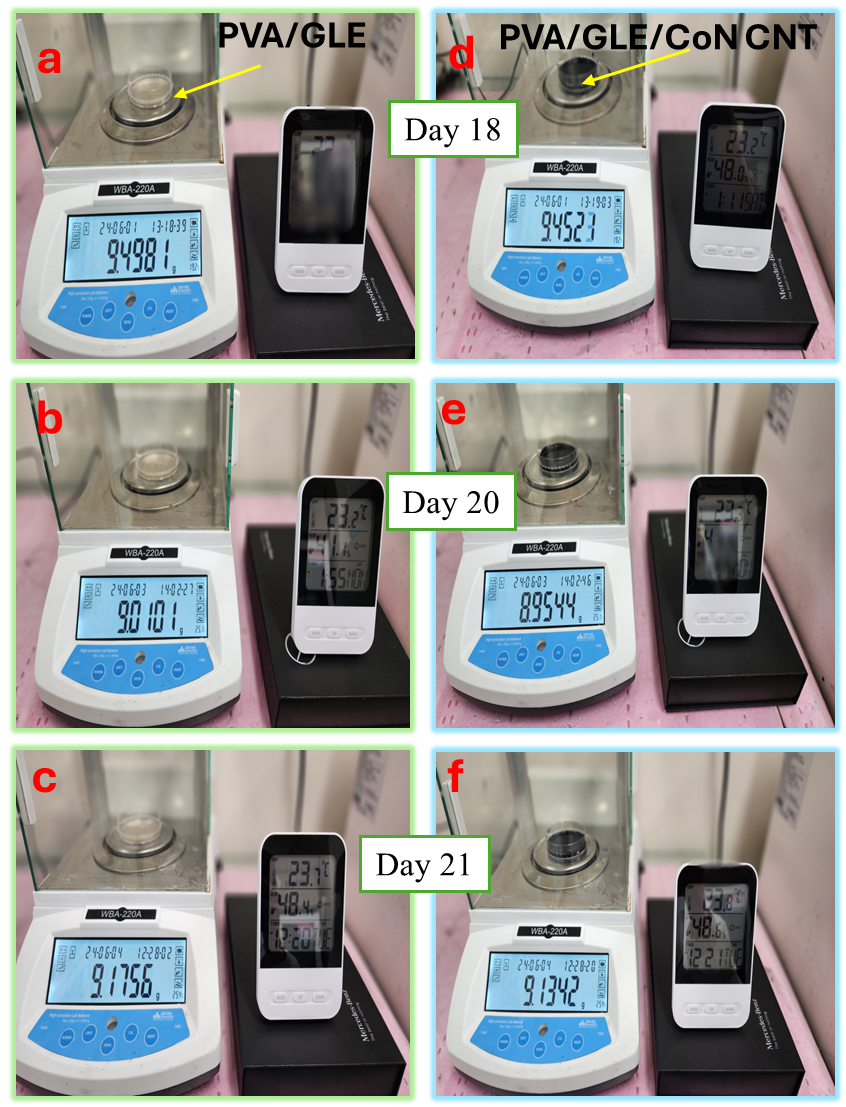


**Fig. S20** Weight retention analysis of **a-c** PVA/GLE, and **d-f** CoN CNT/PVA/GLE organogel under ambient conditions over three days. Images show the mass measurements on Day 18, Day 20, and Day 21


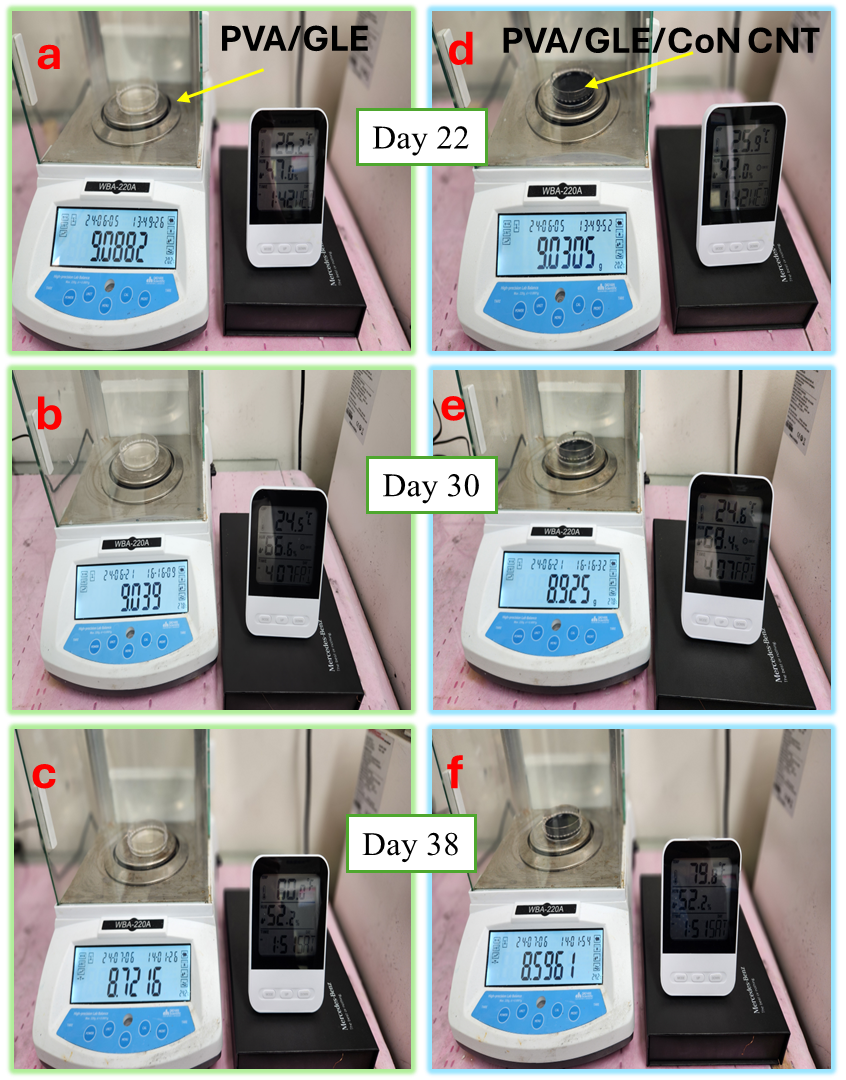


**Fig. S21** Weight retention analysis of **a-c** PVA/GLE, and **d-f** CoN CNT/PVA/GLE organogel under ambient conditions over three days. Images show the mass measurements on Day 22, Day 30, and Day 38


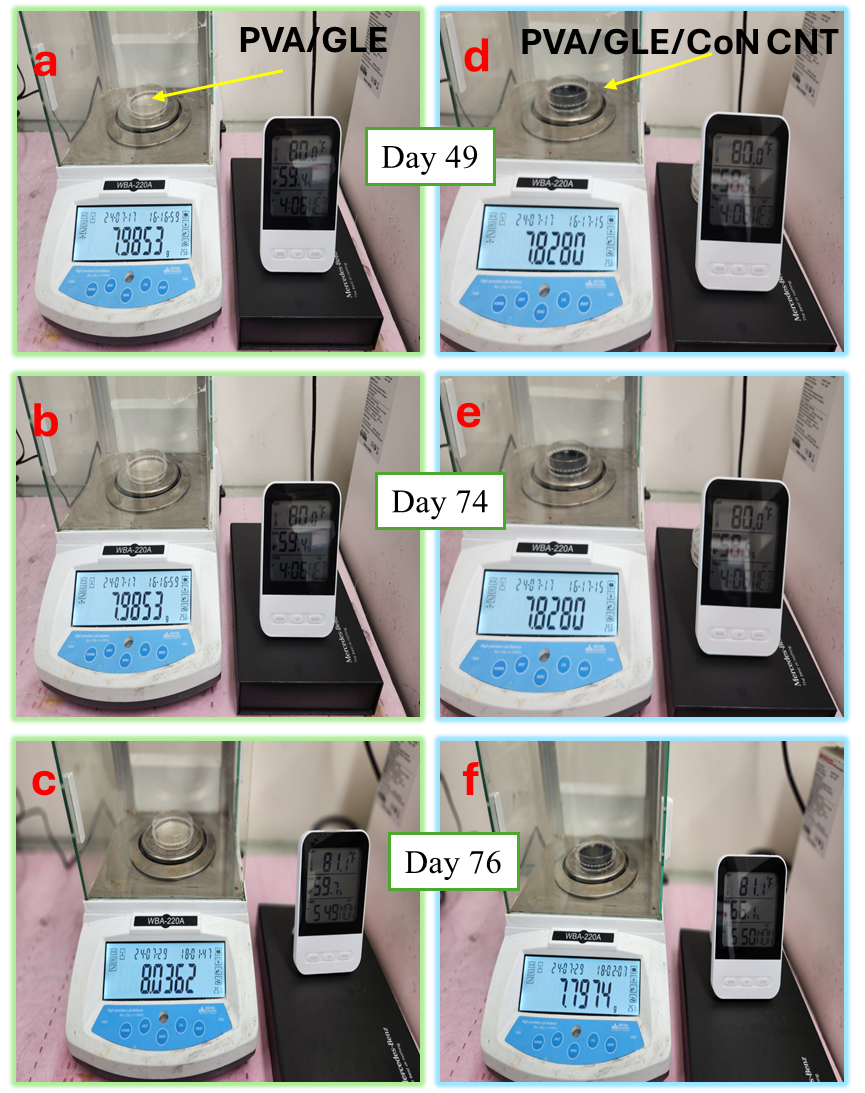


**Fig. S22** Weight retention analysis of **a-c** PVA/GLE, and **d-f** CoN CNT/PVA/GLE organogel under ambient conditions over three days. Images show the mass measurements on Day 49, Day 74, and Day 76


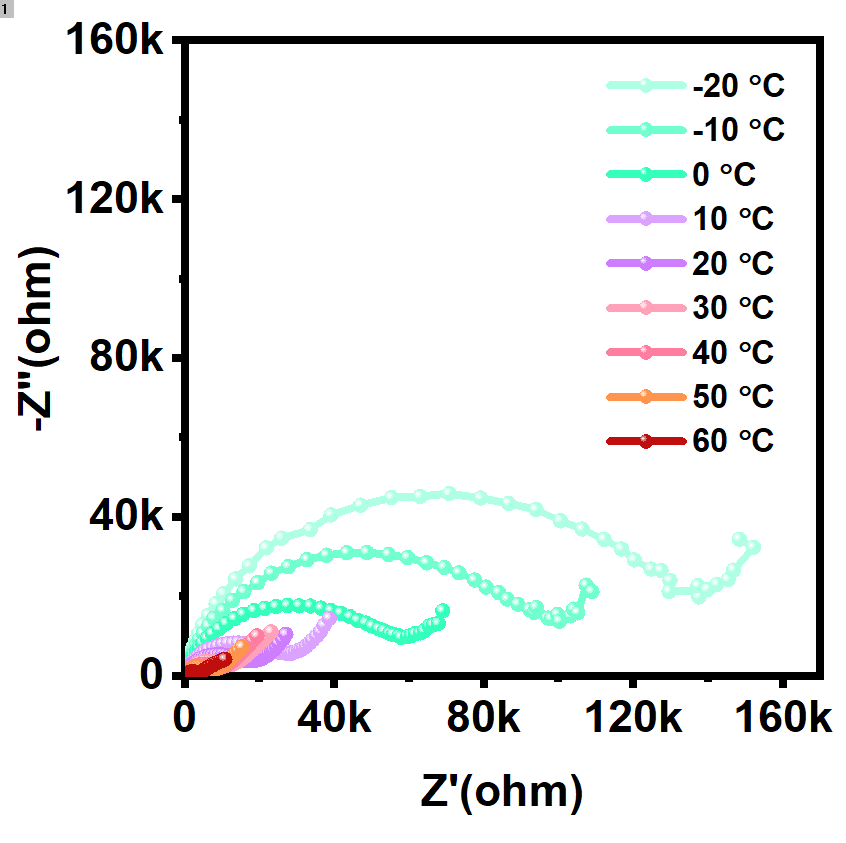


**Fig. S23** Nyquist plot from -20 ºC to 60 ºC for PVA/GLE organogel

The impedance of PVA/GLE is much higher compared to CoN CNT/PVA/GLE organogel. Higher temperatures improve the conductivity of the PVA/GLE material, but it remains significantly more resistive than CoN CNT PVA/GLE organogel.

**
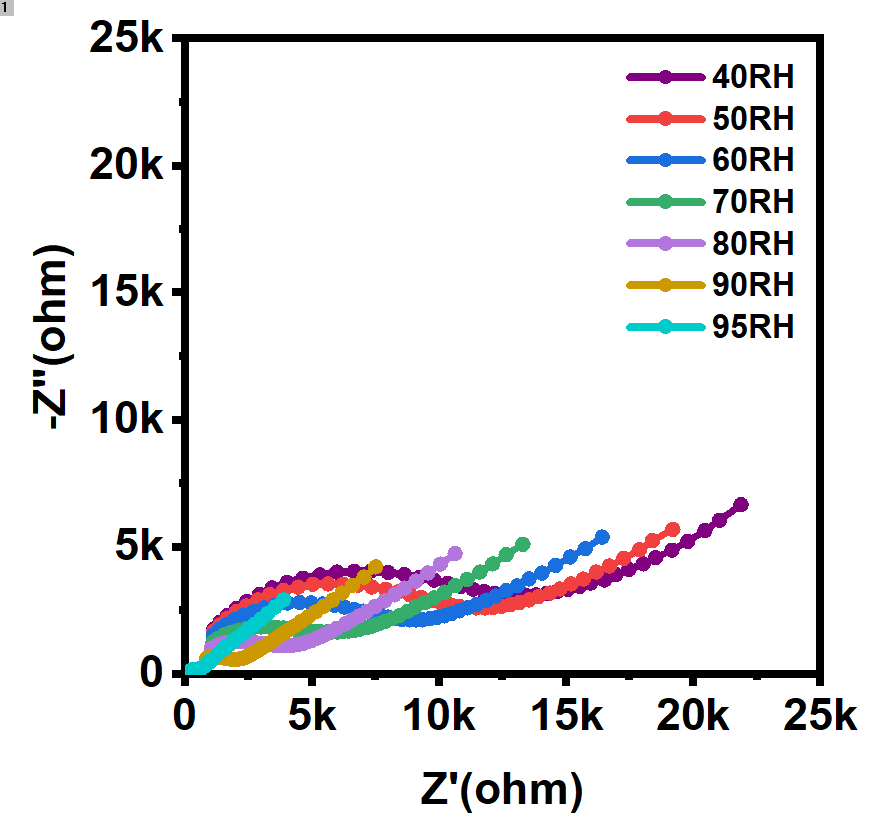
**

**Fig. S24** Nyquist plot from 40% RH to 95% RH for PVA/GLE organogel

**Table S2** Nyquist plots fitting parameter

| CoN CNT/PVA/GLE | | | | | |
| --- | --- | --- | --- | --- | --- |
| Sr. No. | **RH** | **R_s_ (ohm)** | **R_p_ (k-ohm)** | **W**  **Y_0_ (μMho*S^1/2^)** | **CPE**  **Y_0_ (nMho*S^N^), N** |
| 1 | 40 | 642 | 1.2 | 5.12 | 8.99, 0.818 |
| 2 | 50 | 684 | 1.16 | 4.97 | 1.22, 0.926 |
| 3 | 60 | 783 | 1.23 | 4.20 | 0.567, 0.969 |
| 4 | 70 | 857 | 1.32 | 4.71 | 0.264, 1.02 |
| 5 | 80 | 720 | 1.50 | 4.20 | 0.344, 0.979 |
| 6 | 90 | 720 | 1.42 | 4.20 | 0.324, 0.987 |
| 7 | 95 | 998 | 0.998 | 5.96 | 0.334, 1.02 |

**Table S3** Nyquist plots fitting parameter

| PVA/GLE | | | | | |
| --- | --- | --- | --- | --- | --- |
| Sr. No. | **RH** | **R_s_ (ohm)** | **R_p_ (k-ohm)** | **W**  **Y_0_ (μMho*S^1/2^)** | **CPE**  **Y_0_ (nMho*S^N^), N** |
| 1 | 40 | 835 | 12.0 | 3.15 | 2.63, 0.788 |
| 2 | 50 | 577 | 10.9 | 3.17 | 3.92, 0.758 |
| 3 | 60 | 630 | 8.44 | 3.92 | 3.97, 0.765 |
| 4 | 70 | 508 | 6.08 | 4.22 | 10.4, 0.709 |
| 5 | 80 | 455 | 4.09 | 4.65 | 13.4, 0.702 |
| 6 | 90 | 350 | 2.05 | 5.41 | 25.5, 0.679 |
| 7 | 95 | 80 | 820 | 7.33 | 1.84, 0.479 |


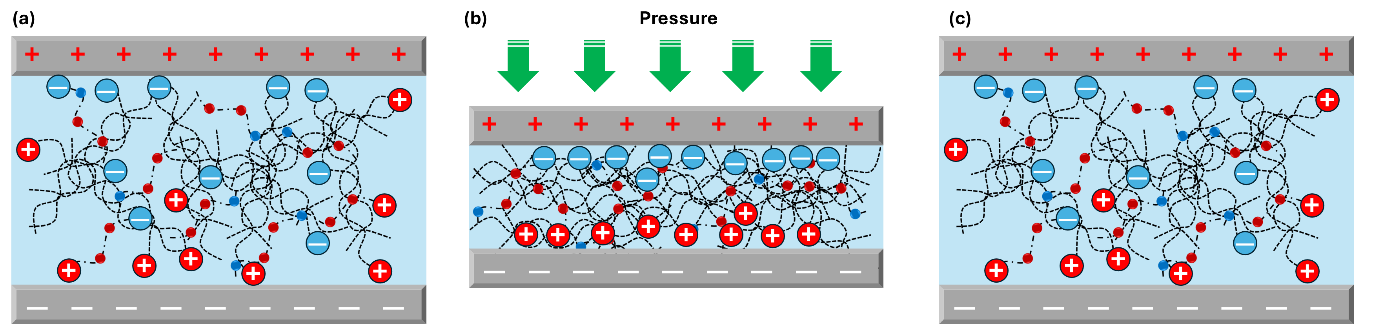


**Fig. S25 a-c** Pressure sensing mechanism


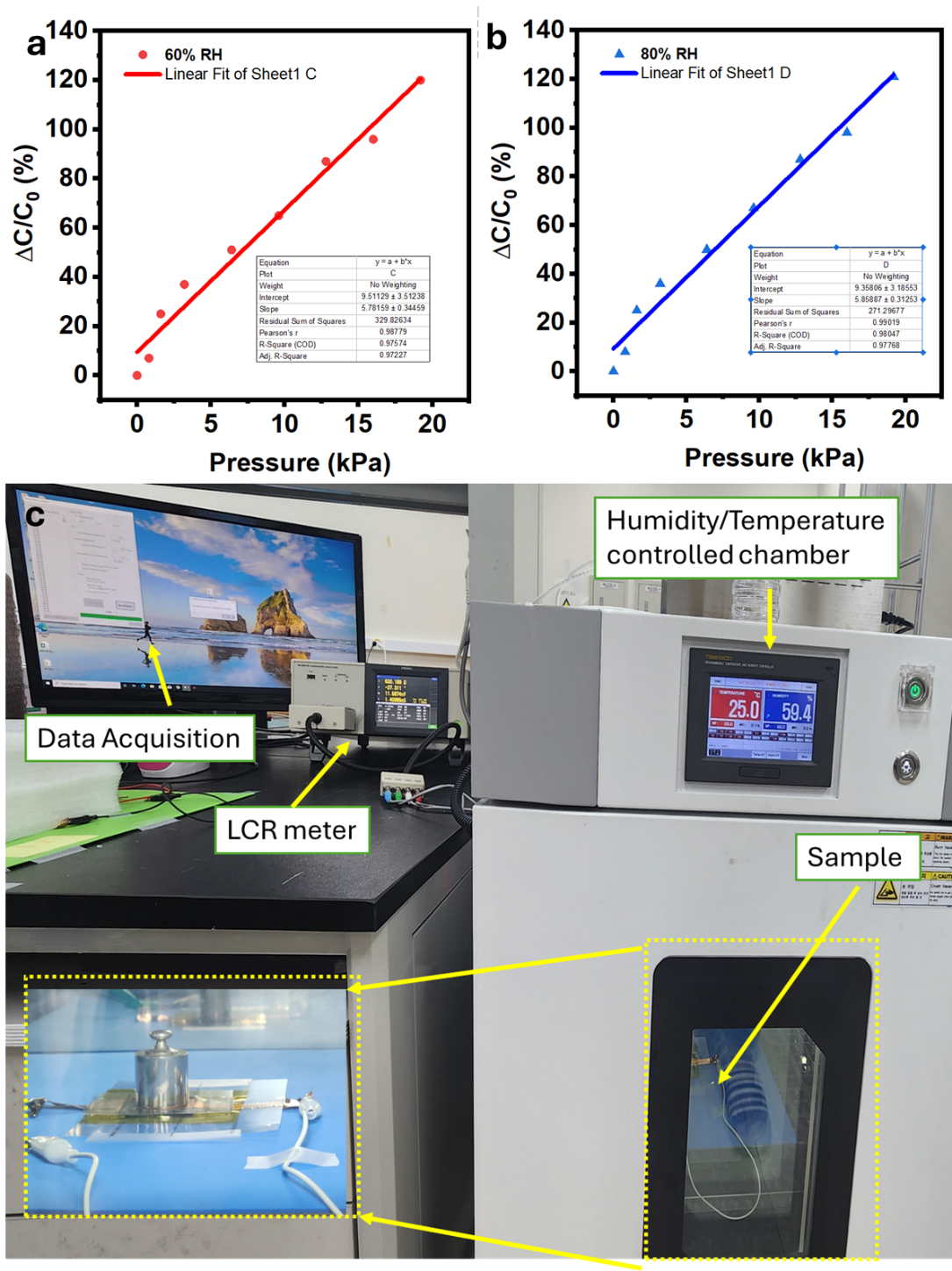


**Fig. S26** Capacitive response plot at **a** 60% RH, **b** 80% RH. Optical images of the pressure sensing set up in humid conditions


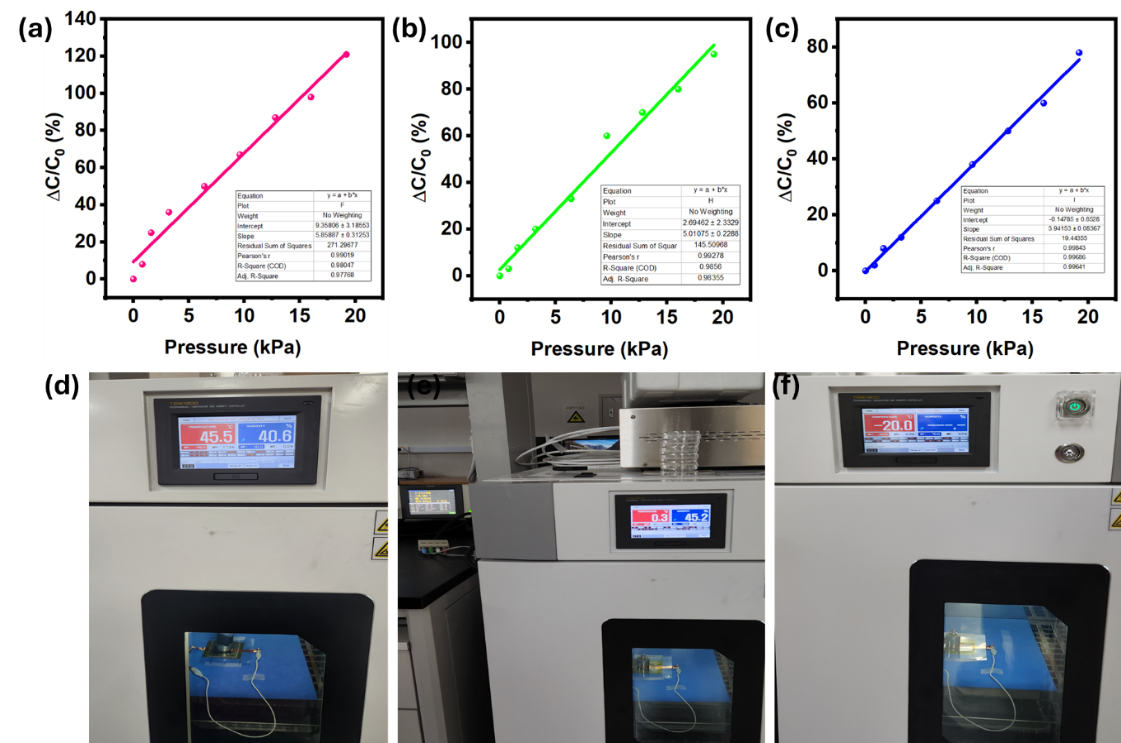


**Fig. S27** Sensitivity plot at **a** 45 °C, **b** 0 °C, and **c** -20 °C. **d-f** respective optical images of the sensing setup


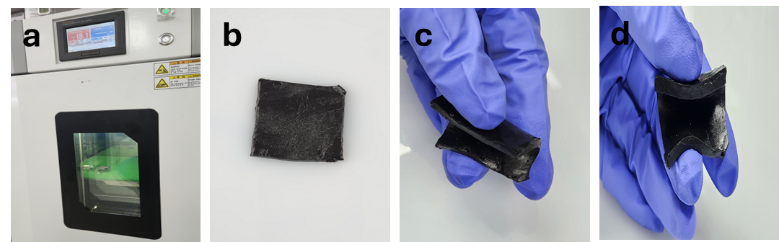


**Fig. S28** **a** organogel stored at -20 ºC, **b** Photograph of the CoN CNT/PVA/GLE organogel, **c-d** in bending condition exhibiting flexibility


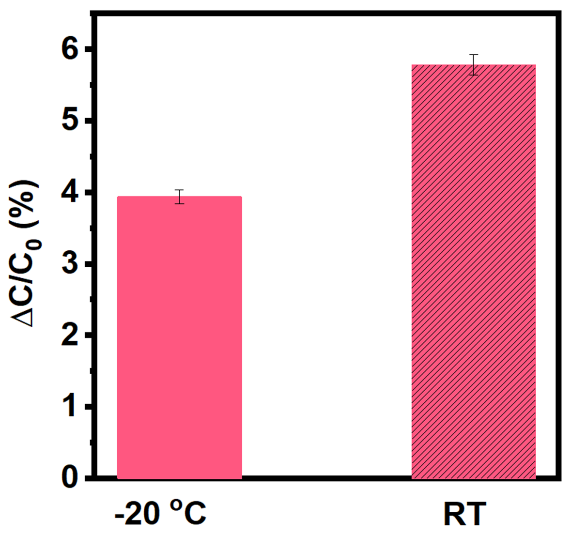


**Fig. S29** Temperature endurance of organogel

**
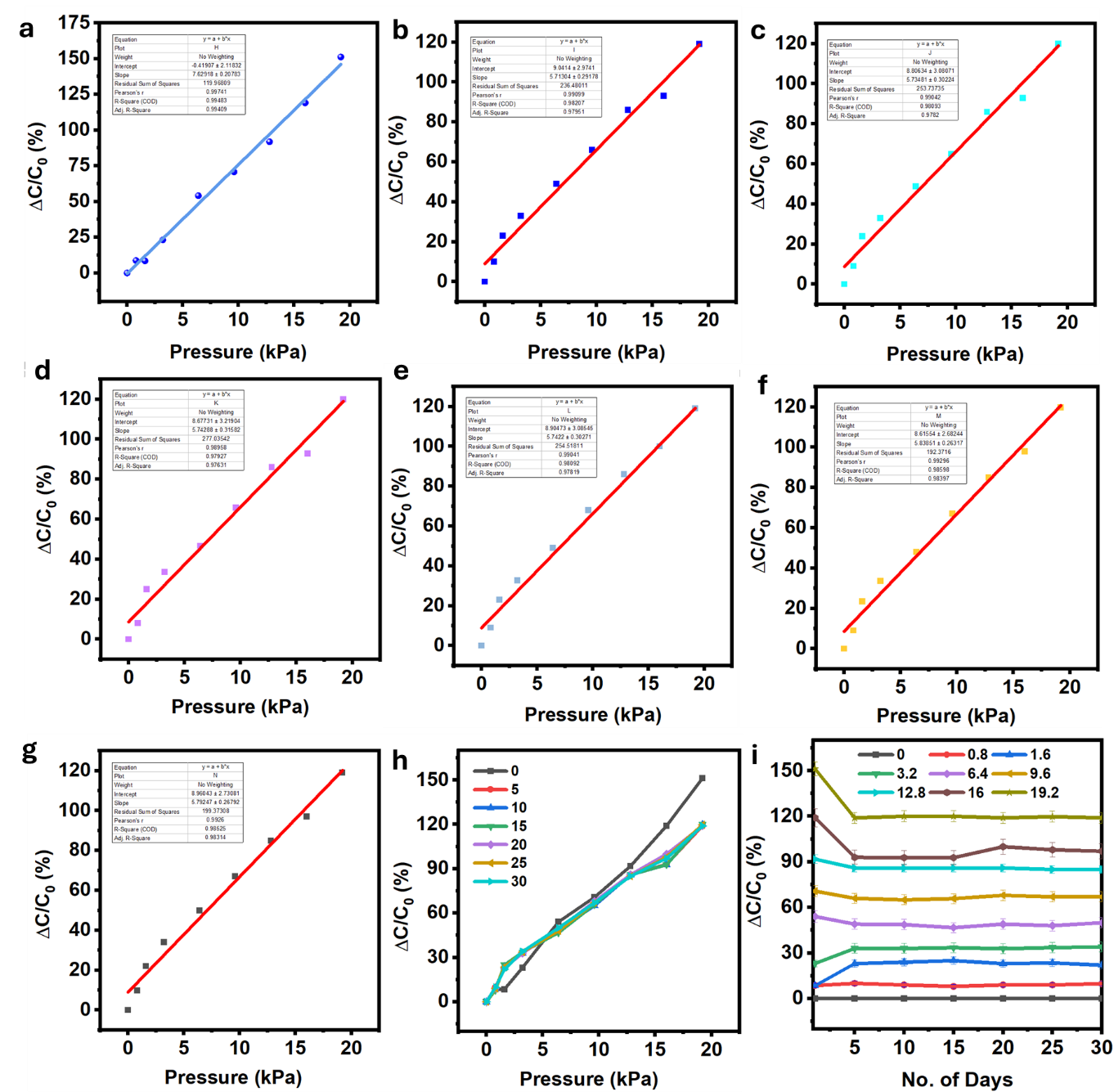
**

**Fig. S30** Capacitive response to pressure sensing in the detection range 0 – 20 kPa **a** Day 1, **b** Day 5, **c** Day 10, **d** Day 15, **e** Day 20, **f** Day 25, **g** Day 30, **h** cumulative capacitive response over a period of one-month, **i** long term stability plot

**Table S4** Summary of the sensitivity data for the CoN CNT/PVA/GEL organogel

| Sr. No. | Days | Sensitivity (%) | Linearity Coefficient (r^2^) |
| --- | --- | --- | --- |
| 1 | 1 | 7.62 | 0.994 |
| 2 | 5 | 5.71 | 0.979 |
| 3 | 10 | 5.73 | 0.978 |
| 4 | 15 | 5.74 | 0.976 |
| 5 | 20 | 5.74 | 0.978 |
| 6 | 25 | 5.83 | 0.983 |
| 7 | 30 | 5.79 | 0.983 |

**
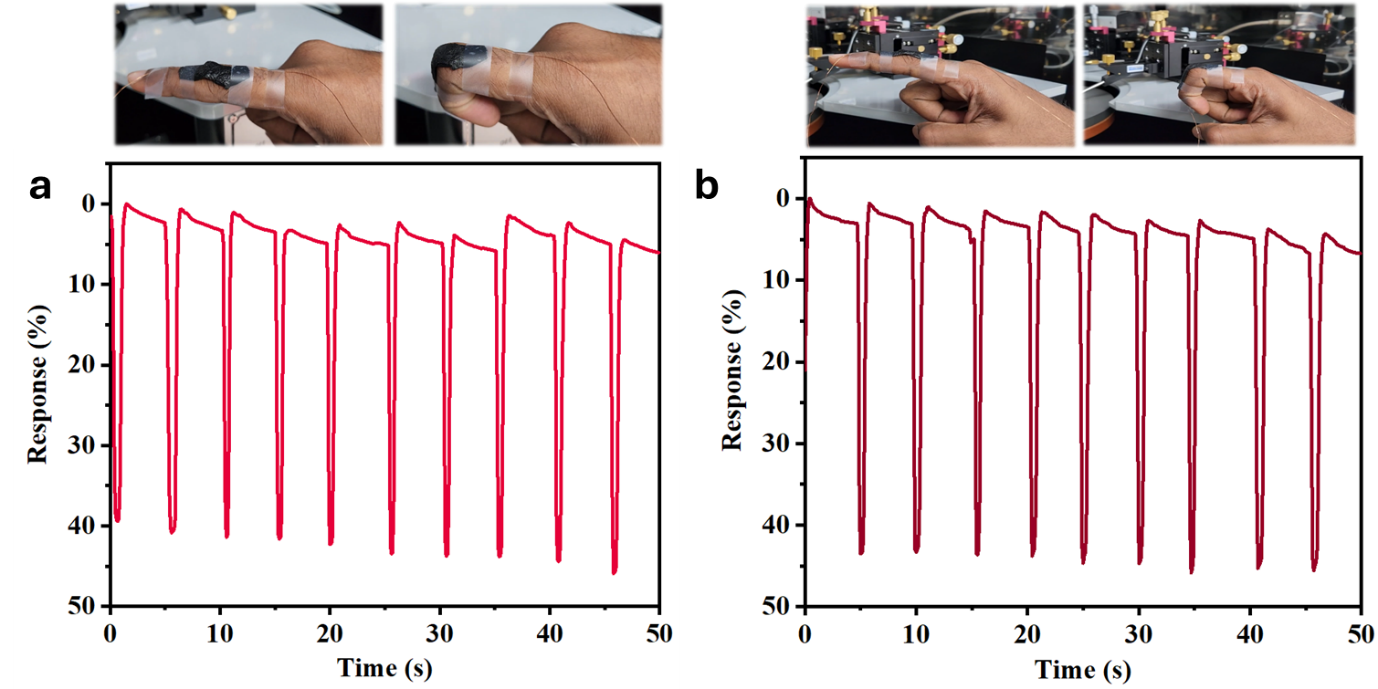
**

**Fig. S31** CoN CNT/PVA/GLE sensor response to finger bending **a** as prepared, **b** after 1 week


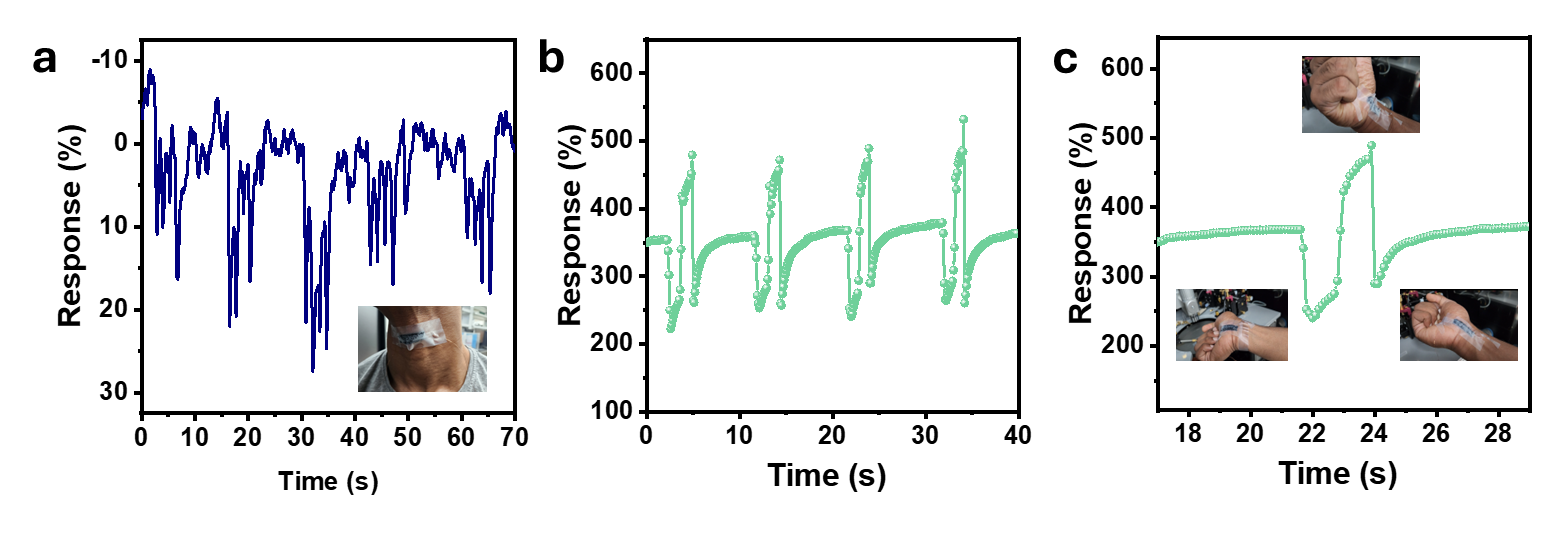


**Fig. S32** The response characteristics while speaking the letters 'N', 'M', 'S,' and 'L' respectively


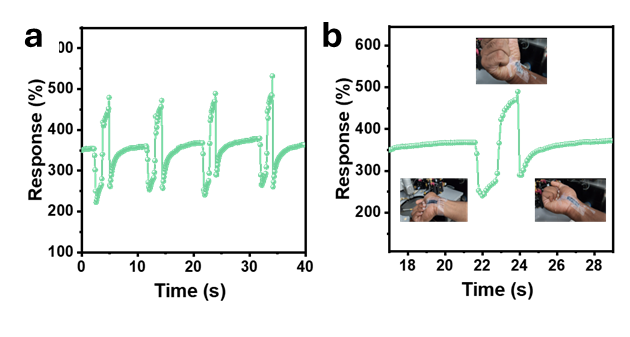


**Fig. S33** **a** Response characteristics for the downward and upward movement of the wrist. **b** magnified version showcasing an individual wrist bending cycle


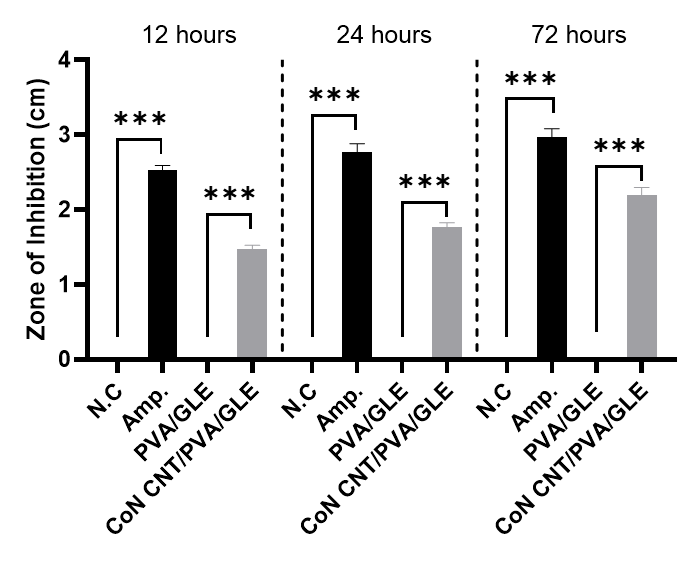


**Fig S34** Zone of inhibition for *E. coli* treated with CoN CNT/PVA/GLE over 3 days. The bar graph presents the mean zone of inhibition (cm) against *Escherichia coli* observed at 12-, 24-, and 72 h following treatment. N.C (Normal Control); PBS, Amp.; Ampicillin (10µg/ml). Independent t-tests were used to compare the zone of inhibition between the normal control and antibiotic drug i.e. ampicillin, and between PVA/GLE and CoN CNT/PVA/GLE treated groups. All values are means ± SD, n=3. ***p<0.0001

**Table S5** Zone of Inhibition (ZOI) measurement details for *E. coli* treated with CoN CNT/PVA/GLE over 3 days

|  | 24hr | | | | 48hr | | | | | 72hr | | | | |
| --- | --- | --- | --- | --- | --- | --- | --- | --- | --- | --- | --- | --- | --- | --- |
|  | N.C | Amp. | PVA/GLE | CoN CNT/ PVA/GLE | | N.C | Amp. | PVA/GLE | CoN CNT/ PVA/GLE | | N.C | Amp. | PVA/GLE | CoN CNT/ PVA/GLE |
| ZOI (cm) 1 | 0 | 2.5 | 0 | 1.4 | | 0 | 2.7 | 0 | 1.7 | | 0 | 2.9 | 0 | 2.2 |
| ZOI (cm) 2 | 0 | 2.5 | 0 | 1.5 | | 0 | 2.9 | 0 | 1.8 | | 0 | 3.1 | 0 | 2.3 |
| ZOI (cm) 3 | 0 | 2.5 | 0 | 1.5 | | 0 | 2.7 | 0 | 1.8 | | 0 | 2.9 | 0 | 2.1 |


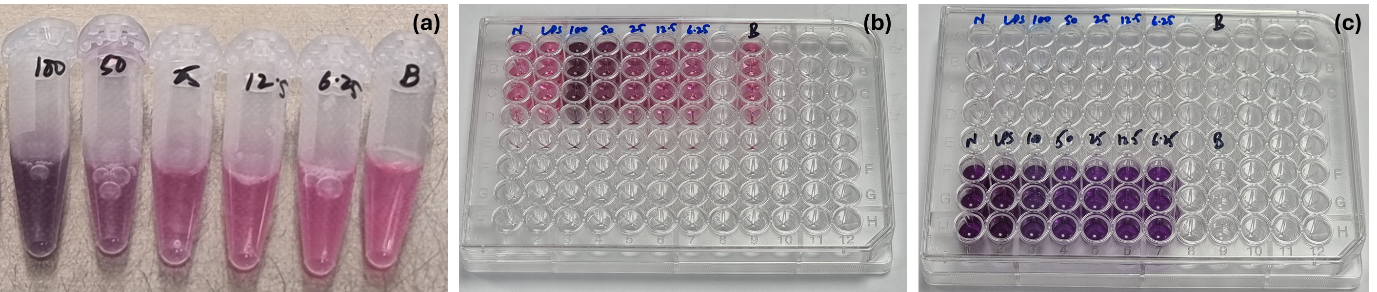


**Fig S35** **a** Samples preparation in DMEM media, **b** Treatment of RAW264.7 cells with samples in a 96-well plate, **c** Process of MTT assay

**Cytotoxicity Test:**

**Table S6** Absorbance taken at 24 h after treatment of RAW264.7 cells with different concentrations of CoN CNT for MTT assay

| Absorbance at 24 h after treatment with CoN CNT concentrations | | | | | | |
| --- | --- | --- | --- | --- | --- | --- |
| Nil | LPS (1µg/ml) | 100 (µg/ml) | 50 (µg/ml) | 25 (µg/ml) | 12.5 (µg/ml) | 6.25 (µg/ml) |
| 3.6767 | 3.201 | 1.7967 | 2.3938 | 2.509 | 2.707 | 2.896 |
| 3.9807 | 2.8022 | 1.7152 | 2.0339 | 2.3496 | 2.6498 | 2.8441 |
| 3.7157 | 3.6295 | 1.5544 | 2.2388 | 2.6093 | 2.7959 | 3.2499 |


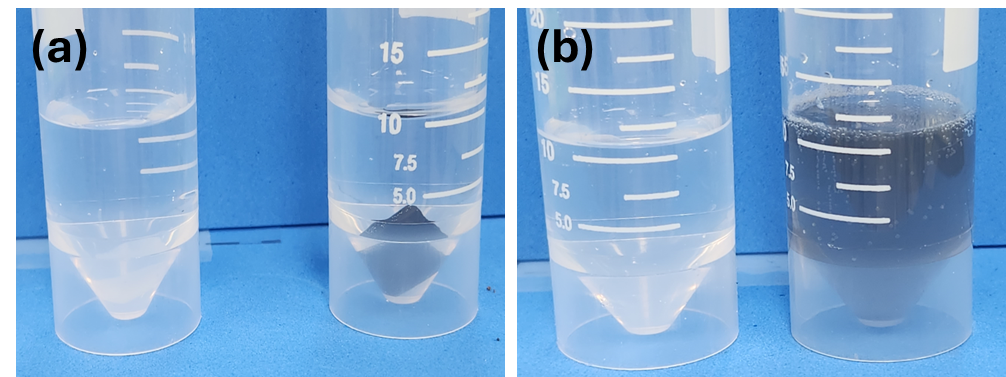


**Fig. S36** PVA/GLE and CoN CNT/PVA/GLE **a** before degradation, **b** after degradation in oxidative media (H_2_O_2_)

**Table S7** Environmental Comparison of CoN-CNT Synthesis Routes

| Parameter | Conventional CVD Method | This Work (Solid State Route) |
| --- | --- | --- |
| Carbon Source | Toxic hydrocarbons (CH₄, C₂H₂) | Melamine (non-volatile, solid organic source) |
| Catalyst Requirement | Requires Fe, Ni, Co metals | Co(NO₃)₃·6H₂O (homogeneous precursor) |
| Reaction Atmosphere | Vacuum, inert/reducing gas flows (H₂, Ar) | Ambient air + Argon (no vacuum) |
| Temperature | >900 °C | 550 °C & 850 °C (lower, two-step) |
| Energy & Safety | High energy, flammable gases | Moderate energy, safer lab setup |
| Environmental Risk | Catalyst residues, gas emissions | Minimal waste; no toxic gases |
| Cost & Scalability | Capital intensive | Low-cost, batch-scalable process |
| End-of-Life | CNTs persistent in soil/water | CoN-CNT remains embedded in degradable gel |

**
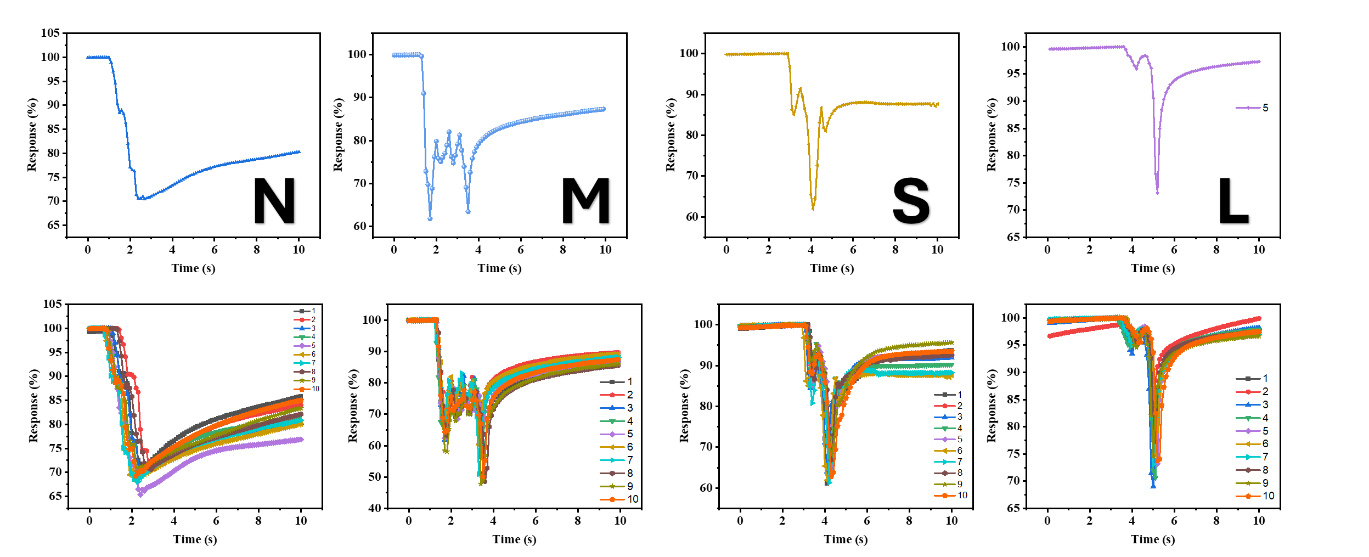
**

**Fig. S37** Patterns of writing the letter ‘N’, ‘M’, ‘S’ and ‘L’ along with response for 10 cycles


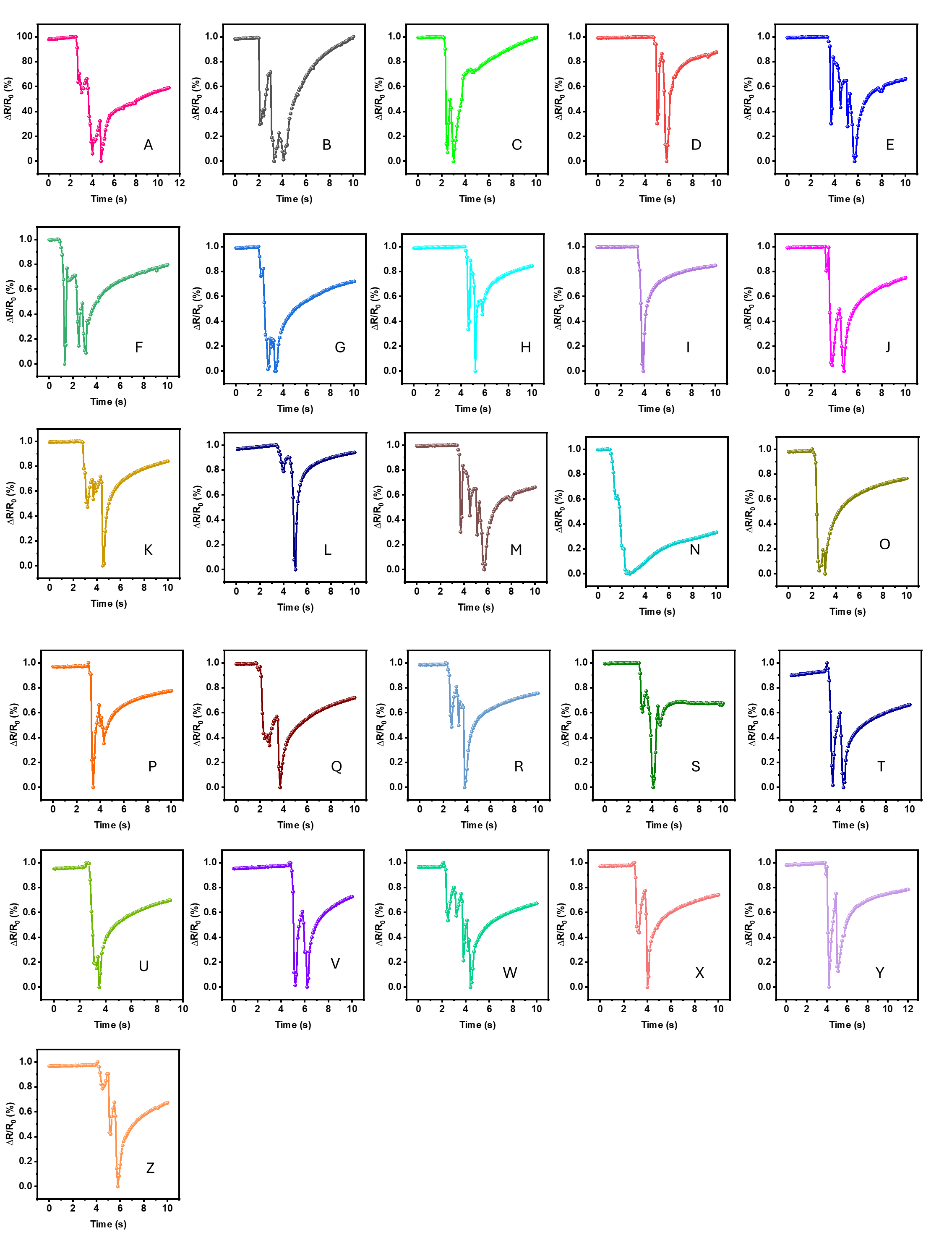


**Fig. S38** Resistive response for English alphabet A-Z


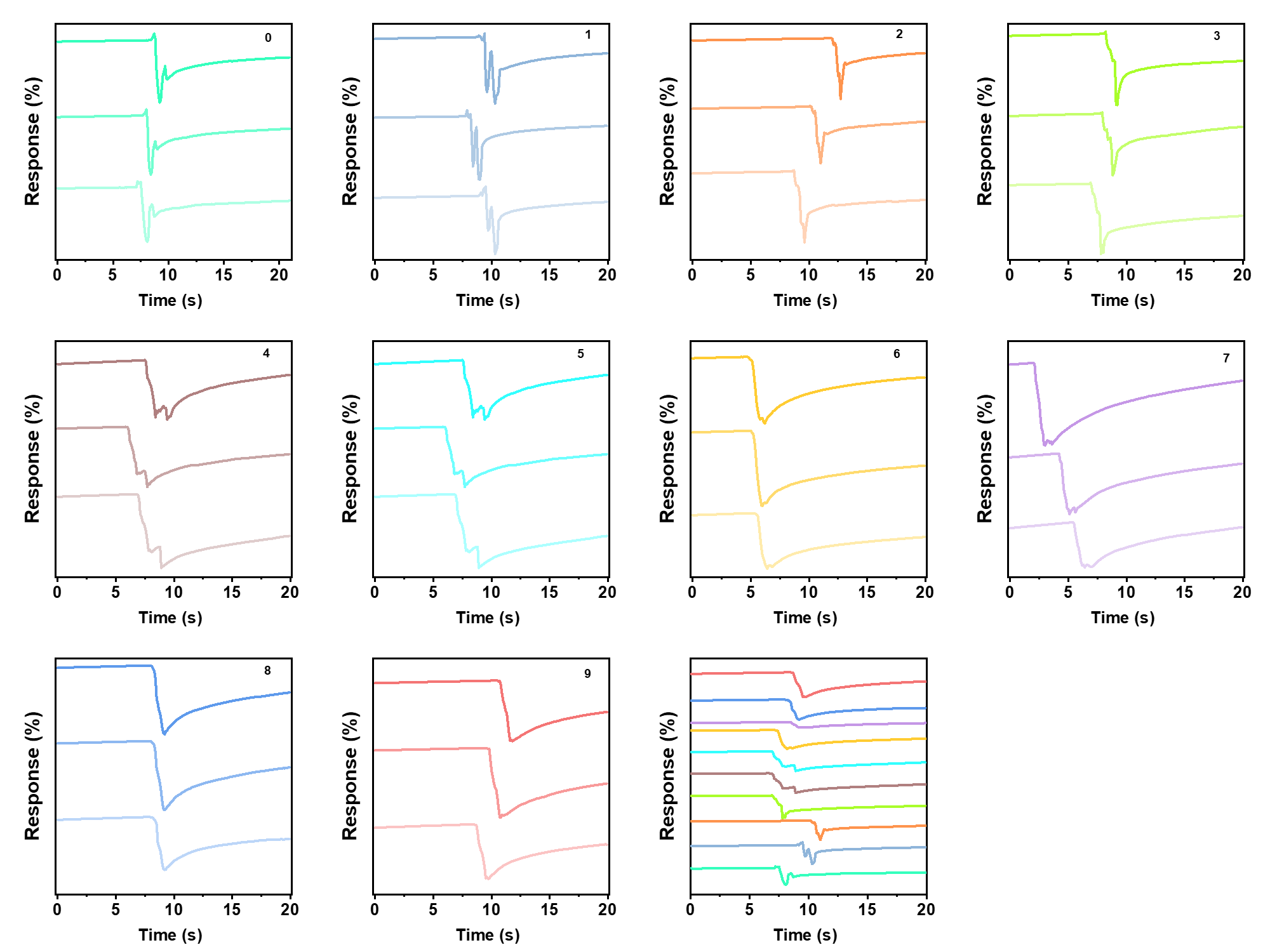


**Fig. S39** Resistive response for numerals 0-9

**Table S8** Number of total, training, and testing samples for each letter (L, M, N, and S) used in the classification models

| Letter | Total Samples | Training Samples | Testing Samples |
| --- | --- | --- | --- |
| L | 1507 | 1205 | 302 |
| M | 1606 | 1285 | 321 |
| N | 1486 | 1189 | 297 |
| S | 1725 | 1380 | 345 |

**Table S9** Comparing the performance of various classifier models

| Classification Model | Accuracy | Precision | Recall | F1-score |
| --- | --- | --- | --- | --- |
| 1D-CNN-LSTM | 0.8656 | 0.8681 | 0.8656 | 0.8650 |
| Stacking Model | 0.9810 | 0.9811 | 0.9810 | 0.9810 |
| XGBoost | **0.9826** | **0.9827** | **0.9826** | **0.9825** |


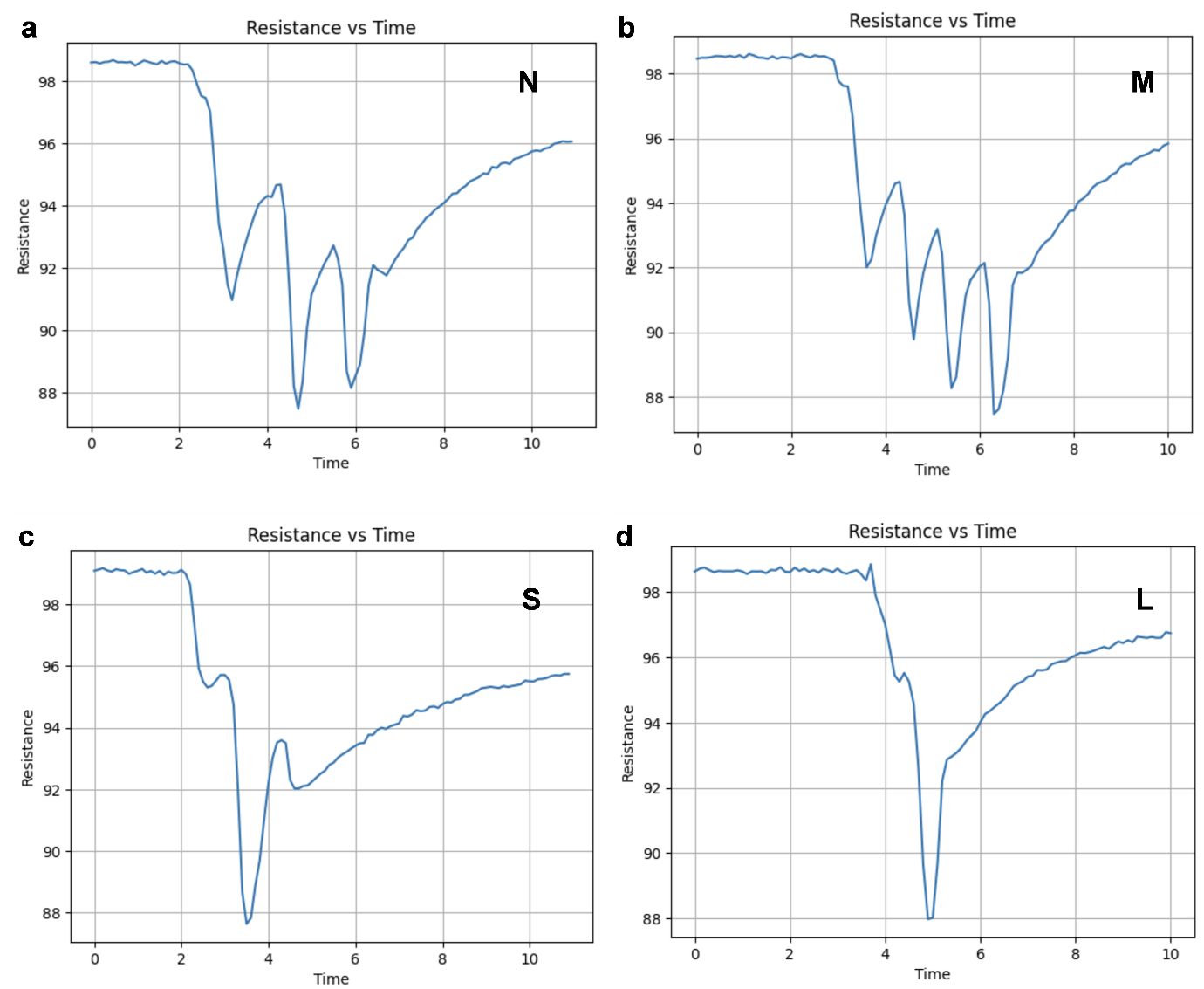


**Fig. S40** Resistance signal for writing variability-such as changes in writing speed and pressure
